# Targeting cathepsin S promotes activation of OLF1-BDNF/TrkB axis to enhance cognitive function

**DOI:** 10.1186/s12929-024-01037-2

**Published:** 2024-05-09

**Authors:** Hao-Wei Lee, Szu-Jung Chen, Kuen-Jer Tsai, Kuei-Sen Hsu, Yi-Fan Chen, Chih-Hua Chang, Hsiao-Han Lin, Wen-Yun Hsueh, Hsing-Pang Hsieh, Yueh-Feng Lee, Huai-Chueh Chiang, Jang-Yang Chang

**Affiliations:** 1 Institute of Biotechnology and Pharmaceutical Research, National Health Research Institutes, Zhunan, Taiwan; 2 Taipei Cancer Center, TMU Research Center of Cancer Translational Medicine, Taipei Medical University Hospital, College of Medicine, Taipei Medical University, No. 252, Wuxing St., Xinyi Dist., Taipei, 110301 Taiwan (R.O.C.); 3 Institute of Clinical Medicine, College of Medicine, National Cheng Kung University, Tainan, Taiwan; 4 Institute of Basic Medical Science, College of Medicine, National Cheng Kung University, Tainan, Taiwan; 5 Department of Pharmacology, College of Medicine, National Cheng Kung University, Tainan, Taiwan; 6 Immunology Research Center, National Health Research Institutes, Zhunan, Taiwan; 7 Department of Biotechnology and Bioindustry Sciences, National Cheng Kung University, Tainan, Taiwan; 8 Research Center of Clinical Medicine, National Cheng Kung University Hospital, College of Medicine, National Cheng Kung University, Tainan, Taiwan

**Keywords:** Cathepsin S, Cognitive function, OLF-1, Brain-derived neurotrophic factor

## Abstract

**Background:**

Cathepsin S (CTSS) is a cysteine protease that played diverse roles in immunity, tumor metastasis, aging and other pathological alterations. At the cellular level, increased CTSS levels have been associated with the secretion of pro-inflammatory cytokines and disrupted the homeostasis of Ca^2+^ flux. Once CTSS was suppressed, elevated levels of anti-inflammatory cytokines and changes of Ca^2+^ influx were observed. These findings have inspired us to explore the potential role of CTSS on cognitive functions.

**Methods:**

We conducted classic Y-maze and Barnes Maze tests to assess the spatial and working memory of *Ctss*^*−/−*^ mice, *Ctss*^+*/*+^ mice and *Ctss*^+*/*+^ mice injected with the CTSS inhibitor (RJW-58). Ex vivo analyses including long-term potentiation (LTP), Golgi staining, immunofluorescence staining of sectioned whole brain tissues obtained from experimental animals were conducted. Furthermore, molecular studies were carried out using cultured HT-22 cell line and primary cortical neurons that treated with RJW-58 to comprehensively assess the gene and protein expressions.

**Results:**

Our findings reported that targeting cathepsin S (CTSS) yields improvements in cognitive function, enhancing both working and spatial memory in behavior models. Ex vivo studies showed elevated levels of long-term potentiation levels and increased synaptic complexity. Microarray analysis demonstrated that brain-derived neurotrophic factor (BDNF) was upregulated when CTSS was knocked down by using siRNA. Moreover, the pharmacological blockade of the CTSS enzymatic activity promoted BDNF expression in a dose- and time-dependent manner. Notably, the inhibition of CTSS was associated with increased neurogenesis in the murine dentate gyrus. These results suggested a promising role of CTSS modulation in cognitive enhancement and neurogenesis.

**Conclusion:**

Our findings suggest a critical role of CTSS in the regulation of cognitive function by modulating the Ca^2+^ influx, leading to enhanced activation of the BDNF/TrkB axis. Our study may provide a novel strategy for improving cognitive function by targeting CTSS.

**Supplementary Information:**

The online version contains supplementary material available at 10.1186/s12929-024-01037-2.

## Background

With the increasing older population globally, cognitive impairment has become a critical problem. Cognitive impairment affects approximately 19% of the global population and is characterized by symptoms such as memory loss, learning difficulties, and loss of the ability to concentrate on a task [1]. Etiological studies on cognitive impairment have suggested that vascular conditions, stroke, neurodegenerative diseases, and aging are predominant factors that increase the risks of dementia and mortality, especially in the older population [2, 3]. Thus, an innovative therapeutic strategy should be developed to address the increasing incidence of cognitive impairment. In the psychology, “cognitive function” is a broad term that encompasses the integration of mental processes involved in acquiring knowledge, manipulating information, and reasoning. Complicated cognitive behavior involves multifaceted domains, including memory and learning [4]. However, at the tissue and molecular levels, long-lasting functional and anatomical changes occurring in synaptic structures in response to environmental stimuli may lead to alter cognitive function [5, 6]. For this reason, the remodeling of synaptic complexity through diverse approaches is warranted.

Cathepsin S (CTSS) is a lysosomal cysteine protease that belongs to papain superfamily. In physiological conditions, CTSS is synthesized in an inactive form and is activated by its pro-peptide within the lysosome particularly in the antigen presenting cells [7–9]. Once activated, CTSS exhibits proteolytic activity at neutral pH, enabling it to degrade various components of the extracellular matrix such as laminin, collagens, and elastin in lysosomes [7, 10, 11]. Once lysosomal injury, the release of autophagosomes results in the extracellular secretion of CTSS. Subsequently, CTSS enzymatically cleaves the extracellular matrix, further contributing to the remodeling and plasticity of microenvironments. Recently, CTSS has been reported to be associated with immunity [8, 9], tumor metastasis [12, 13], cardiovascular disorders [14, 15], psoriasis [16], neurodegenerative disorders [17], peripheral neuropathy [18], and aging [19, 20]. Our previous study demonstrated that targeting CTSS suppressed proinflammatory cytokine secretion and promoted anti-inflammatory cytokine secretion, protecting neurons from oxaliplatin-induced peripheral neuropathy, indicating that the inhibition of CTSS reduced tissue inflammation [18]. The maintenance of Ca^2+^ homeostasis is another crucial function of CTSS. By interfering with stromal interaction molecule 1 (STIM1) trafficking, CTSS prevents store operated Ca^2+^ entry (SOCE), thus dysregulating Ca^2+^ influx [21]. However, the role of CTSS in the regulation of cognitive functions has never been explored.

Brain-derived neurotrophic factor (BDNF) belongs to the neurotrophic factor family, which is mainly synthesized in the brain and is widely distributed in both neuronal and non-neuronal cells [22]. BDNF has been implicated in neurogenesis, neuronal survival [23], differentiation [24], degeneration [25, 26], neuronal plasticity [27], and memory formation [28, 29]. BDNF is considered a crucial neurotrophic factor for mitigating neuropsychiatry disorders [30, 31]. The binding of BDNF to tropomyosin receptor kinase B (TrkB) leads to ligand–receptor dimerization and tyrosine residue autophosphorylation in the intracellular domain of TrkB. Subsequently, three well-known signaling cascades, namely Ras-MAPK, PI3K-Akt and PLCγ-Ca^2+^ pathways, are activated following the phosphorylation of different tyrosine residues on TrkB [26, 28]. In addition, cAMP-responsive element binding protein (CREB), a BDNF transcriptional activator, undergoes phosphorylation and promotes BDNF transcription [31]. Low BDNF levels in the brain is associated with the occurrence of various neurological disorders [25]. The current strategies to improve BDNF levels in the brain has been focused on exercise, diet, and anti-depressant use [32]. Unfortunately, none of these strategies have proven to be effective in treating cognitive impairment, especially in the older population. In this study, we offer new option for targeting CTSS, which regulates Ca^2+^ influx switches on the BDNF/TrkB signaling axis in the brain, to improve cognitive functions. We additionally identified the underlying molecular mechanisms.

## Materials and methods

### Animals

We purchased 8–12-week-old adult male wildtype C57BL/6JNarl (*Ctss*^+*/*+^) mice from National Laboratory Animal Center and bred them in the animal center of the National Health Research Institutes (NHRI). Mice were group housed (*N* = 5/cage) in individually ventilated cages under conventional conditions and provided food pellets (LabDiet 5001) and tap water ad libitum. The light–dark cycle is set as 12 h/12 h. All experiments were approved by the Institutional Animal Care and Use Committee of the NHRI (IACUC No.: 110002).

### Generation of Ctss null (Ctss^−/−^ ) strain

The National Laboratory Animal Center (Taiwan) generated *Ctss*^−/−^ mice by using the CRISPR/Cas9 system. A point mutation, TGACTA, was introduced into the CTSS sequence, which includes a stop codon and the SpeI restriction enzyme recognition site, to verify mutations at exon 2 of the *CTSS* gene. Recombinant embryonic stem cells were injected into 161 C57BL/6JNarl blastocysts; 122 of them were transferred into pseudo‐pregnant females, and 28 live pups were born in 2018. Only one *Ctss*^+/−^ female strain was detected through polymerase chain reaction (PCR) genotyping. For recessive phenotypes, an intercross between female *Ctss*^+/−^ mice and male *Ctss*^+*/*−^ led to the generation of *Ctss*^−/−^ mice. The offspring were then backcrossed three times to generate *Ctss*^−/−^ mice (referred to as P0). Subsequently, approximately 150 *Ctss*^*−/−*^ embryos were retrieved from P0 *Ctss*^*−/−*^ mice and preserved in liquid nitrogen. All parental *Ctss*^*−/−*^ mice (referred to as P1) in our study originated from *Ctss*^*−/−*^ frozen embryos obtained from the recessive P0 *Ctss*^*−/−*^ mice. Furthermore, all *Ctss*^*−/−*^ mice utilized in our study were restricted to fewer than two generations (< P2). C*tss*^+*/*+^ C57BL/6JNarl mice were used as controls. Primers used for *Ctss*^−^/^−^ genotyping are listed in Table S2.

### Cell culture

The immortalized mouse hippocampal neuronal cell line HT-22 was purchased from Merck (#SCC129, USA). HT-22 cells were maintained in an expansion medium consisting of high-glucose Dulbecco’s modified Eagle medium (DMEM), 10% fetal bovine serum, and 1 × penicillin/streptomycin/glutamine solution. The culture medium was refreshed every 2 to 3 days. HT-22 cells were trypsinized and plated on another new culture dish when they reached approximately 70% confluence. Cells were incubated at 37°C and humidified under 5% CO_2_. All reagents and chemicals that utilized in this study were listed in Table S3.

### Primary cortical neuron culture

For primary cortical neuron culture, a culture dish was coated with a coating buffer (0.47 g of boric acid, 0.254 g of borax, and 100 mg of poly-D-lysine in 100 ml of dd-H_2_O) for at least 3 h. Then, the coating buffer was discarded. The coated dish was rinsed with dd-H_2_O at least two times and then dried. Postnatal (P0/P1) C57BL/6JNarl (*Ctss*^+/+^) mice were deeply anesthetized with Attane (isoflurane; Panion & BF Biotech Inc.), their frontal and parietal bones were carefully removed, and the cortex was dissected. Subsequently, the arachnoid mater covering the cortex was carefully removed using forceps. The cortical tissues were resuspended in 5 ml of serum-free DMEM/F12 medium, and the mixture was gently pipetted several times to dissociate the cortical tissues. Then, 500 µl of 0.05% trypsin–EDTA was added (medium: trypsin–EDTA = 10:1, v/v). The cortical tissues were enzymatically digested in a 37°C water bath for 15 min. After the enzymatic cleavage step, cell pellets were resuspended into Hank’s balanced salt solution (HBSS) and centrifuged at 1000 rpm for 10 min. Finally, cell pellets were resuspended into the culture medium (1% L-glutamine, 2% B-27 supplement, 1% penicillin–streptomycin in 100 ml of Neurobasal-A medium) and filtered through a 70-μm nylon mesh (Falcon). All reagents and chemicals that utilized in this study were listed in Table S3. Filtered cells were plated on a poly-D-lysine-coated dish. Cultured primary cortical neuron cells were maintained at 37°C and humidified in 5% CO_2_. The culture medium was refreshed every 3 days.

### Compound synthesis and drug administration

The selective CTSS inhibitor RJW-58 (molecular weight = 582.72) was synthesized as described previously [12, 21]. The specificity of RJW-58 has been reported to possess selective properties targeting CTSS [21]. For in vitro assay, RJW-58 was dissolved in dimethyl sulfoxide (DMSO; Sigma) to obtain desired concentrations. For animal studies, RJW-58 was dissolved in an injection buffer consisting of phosphate-buffered saline (PBS), anhydrous ethanol, and cremophor at a volume ratio of 18:1:1. RJW-58 was administered intraperitoneally. The DMSO/Mock groups in respectively in vitro and in vivo experiments were applied equal volume of DMSO and injection buffer without containing any RJW-58. The total volume ratio of DMSO in culture plate is below 0.1% and the injection buffer was injected intraperitoneally with the ratios of 100 μl per 20 g body weight.

### Section preparations and extracellular field potential recording

Mice were anesthetized with isoflurane (Rhodia Organique Fine Ltd) and sacrificed through decapitation. The hippocampus was cut into 400-µm slices by using the Leica VT1200S vibrating blade microtome (Leica Biosystems) in ice-cold artificial cerebrospinal fluid (ACSF), which contained 117 mM NaCl, 4.7 mM KCl, 2.5 mM CaCl_2_, 1.2 mM MgCl_2_, 25 mM NaHCO_3_, 1.2 mM NaH_2_PO_4_, and 11 mM glucose with 95% O_2_/5% CO_2_ at pH 7.4. Before recording, we placed the slices in a resting chamber with oxygenated ACSF for at least 1 h at room temperature. For extracellular field potential recording, slices were transferred into a recording chamber with oxygenated ACSF at rate of 2 to 3 ml/min at 32°C ± 0.5°C. Recording microelectrodes were pulled from the microfiber 1.0-mm capillary tube by using a Brown–Flaming electrode puller (Sutter Instruments) and filled with 1 M NaCl (2 to 3 MΩ resistance). Field excitatory postsynaptic potentials (fEPSPs) were evoked by a bipolar stimulation electrode that was placed at the Schaffer collateral afferents of the CA1 stratum radiatum at 0.033 Hz. The stimulation intensity was set at a level that evoked a response between 30 and 40% of the maximum response. Long-term potentiation (LTP) was induced by two trains of 1-s high-frequency stimulation (100 Hz) at a 20-s interval. The responses were recorded using an Axoclamp-2B amplifier (Molecular Devices), filtered with a low-pass filter at 2 kHz, digitally sampled at 10 kHz, and analyzed using pCLAMP 8.0 software (Molecular Devices). The slopes of fEPSPs were calculated from 20%–70% of the rising phase and normalized with the average of the 10-min baseline value. The percentage change in fEPSPs was averaged from responses recorded during the last 10 min and analyzed using the Mann–Whitney U test. For analyzing the LTP in hippocampus, 3 slices/mouse were analyzed to obtain an average LTP intensity, and totally 10 mice /group were analyzed.

### Golgi staining

To examine the morphology of the spine head of neuron cells, we performed Golgi staining by using the FD Rapid GolgiStain kit (FD NeuroTechnologies Inc.) in accordance with the manufacturer’s guidelines. In brief, the whole brain was immersed in solution mixture (solution A + solution B) and stored in dark for 14 days. Brain tissues were cut into 200-μm-thick slices by using a vibratome (Dosaka, Japan). The sections were rinsed with dd-H_2_O, transferred to 30% sucrose solution, and placed in dark at 4 °C. Subsequently, slides were washed with dd-H_2_O for 1 min and re-immersed into a solution mixture (solution D + solution E) for 15 min. Slides were washed with dd-H_2_O for 1 min and dehydrated by immersing sequentially in gradient ethanol solutions ranging from 50 to 100% twice for 5 min each, followed by a 2-min immersion in xylene. Slides were mounted using five drops of Eukitt (Fluka Analytical) and covered with a cover glass. Dendritic spines were photographed at a high-power view (40 × and 60 ×) by using the Eclipse TE2000 inverted microscope (Nikon Instruments) to count spine numbers and determine dendritic spine density. The total dendritic length and branch numbers were analyzed using NIS-Elements AR software (Nikon Instruments). For analyzing the dendritic spine density in hippocampus, 6 sections were photographed per mouse and the averaged numbers of dendritic spine density was shown as a dot. Total 5 mice/group were used in dendritic spine density quantification.

## Behavior testing

### Y-maze recognition and alternation tests

**Fig. 1:**
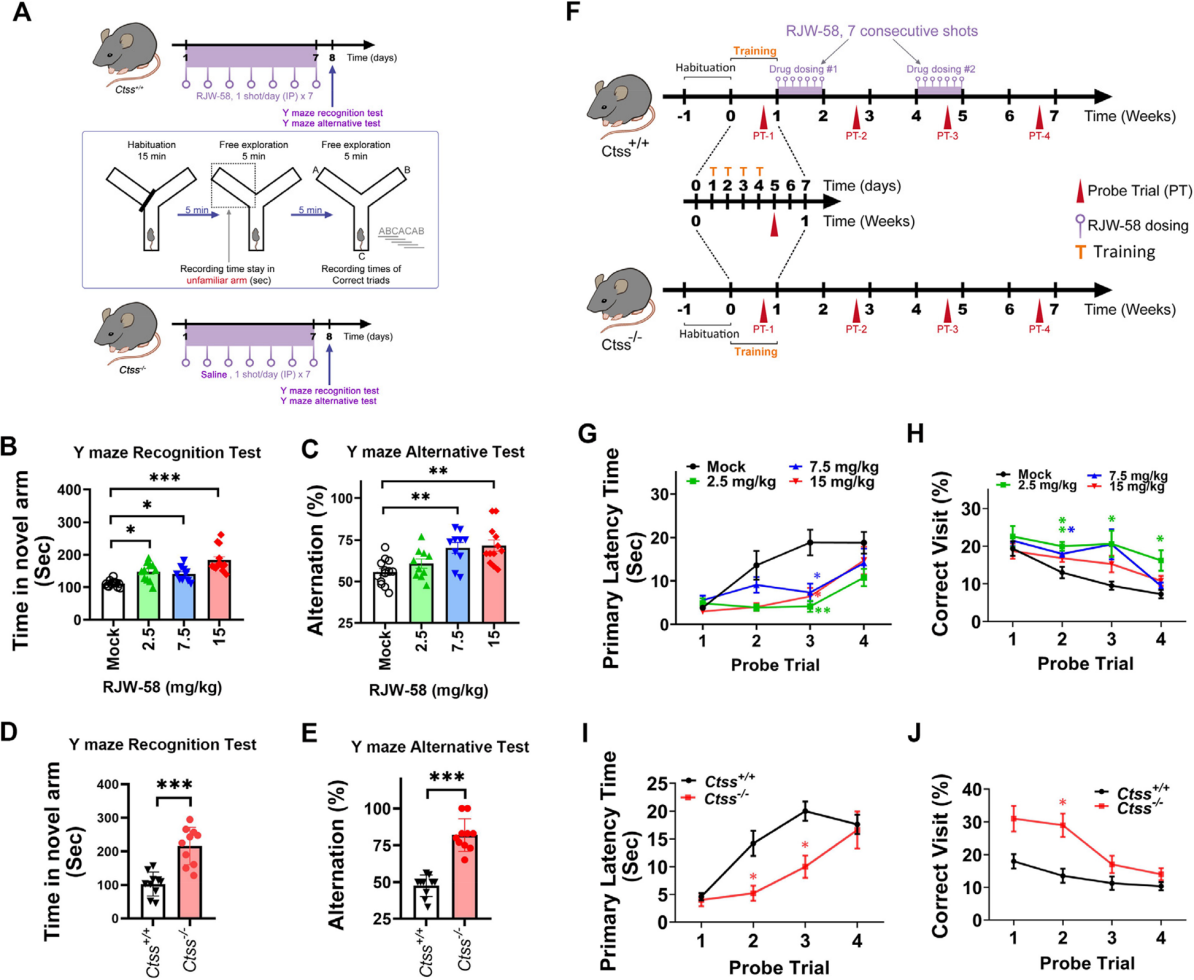
Targeting CTSS enhanced cognitive functions in murine neurological behavior models. **A** Schematic illustrating the timelines of RJW-58 administration and two Y-maze tests. Analyses of spatial and working memory in the Y-maze recognition test (**B**) and alternative test (**C**) when mice received different doses of RJW-58. **D**, **E** Assessment of spatial and working memory in *Ctss* wild-type (*Ctss*^+*/*+^) and knockout (*Ctss*^−/−^) mice (*N* = 10 mice/group) in the Y-maze recognition test (**D**) and alternative test (**E**). **F** Schematic indicating the timeline of RJW-58 dosing and probe trials in the Barnes maze test. Alterations in the primary latency time between different RJW-58-treated groups (**G**) and between *Ctss*^+*/*+^ and *Ctss*^−/−^ mice (**I**). Comparison of the number of correct visits between different RJW-58-treated groups (**H**), and between *Ctss*^+*/*+^ and *Ctss*^−/−^ mice (**J**). Bar charts indicate the mean ± SEM. Asterisks indicate significant differences, Dunn’s multiple comparisons *post-hoc* test in B,and C; Mann–Whitney test in D, and E; Tukey’s multiple comparisons *post-hoc* test in G, and H; Bonferroni’s *post-hoc* test in I, and J, *p < 0.05, **p < 0.01, ***p < 0.001 vs the *Ctss*^+*/*+^ group in D, E, I, and J and the control group in B, C, G, and H

Short-term spatial working memory and exploratory activity were assessed after a 7-day- consecutive RJW-58 administration. To examine the recognition function of RJW-58-treated mice, we performed the recognition test in a three-arm 120º plastic Y-maze. The paradigm is presented in Fig. 1A. In brief, a selected arm was blocked by a transparent insert. In each trial, the mouse was placed in the designated start arm of the maze and allowed to habituate for 15 min. Apart from the blocked arm, the mouse was allowed to explore the maze freely. Then, the mouse was removed from the maze, and a 5-min break was provided before the trial. At the start of the trial, the transparent insert was removed. Each mouse was gently placed at the end of the start arm, facing away from the center. During the trial session that lasted 5 min, the total number of arms entered, and the time spent in an unfamiliar arm were measured and recorded.

An alternation test in the Y-maze was performed after a 5-min break. Each mouse was placed in the central area. The number and order of arm entries were recorded through visual observation within 5 min. Arm entry was defined as the half body (two fore paws) of the mouse entering the arm. The correct triad was defined as the mouse entering all three arms consecutively. For instance, a pattern of A-B-C-A-B consists of 3 alternations (A-B-C, B-C-A, C-A-B); a pattern of A-B-C-B-A consists of only 2 alternations. The alternation rate was calculated by dividing the number of correct triads by the total arm entries minus 2 and then multiplying the result by 100%.

### Barnes maze

A classic Barnes maze (BM) disc was used to assess learning and spatial memory. The testing protocol, extra-maze cue decoration, and escape box setup were based on techniques reported in another study [33], with minor modifications. To determine whether RJW-58 treatment can enhance spatial memory, all enrolled animals underwent a 4-day consecutive training trial before probe trials (PTs) and RJW-58 dosing. The mouse was placed at the center of the maze in a nontransparent cover chamber for 10 s. A 600-lm light bulb and an electric fan were turned on, and they acted as weak stimuli to increase animal motivation during trials. After 10 s, the cover chamber was removed, and the mouse was allowed to explore the entire maze freely for 3 min. Extra-maze cues around the maze were set up as reference hallmarks to help the mouse learn the relative position of the escape box located under the target hole in the maze. A trial ended immediately after the mouse entered the escape box and stayed there for 1 min. Mice that could not locate the target hole during the 3-min free exploration of the maze were guided into the target hole and allowed to stay there for 1 min. Four repeat trials were performed each day during the training phase. During PTs, the escape box was removed, but all other setups were retained. After a 10-s onset period, the mouse explored the maze to locate the target hole within 90 s. The maze was cleaned with 70% ethanol solution between trials. To determine whether RJW-58 improved learning memory, training trials were performed as described above. All enrolled animals were administered RJW-58 at indicated dosages prior to training trials (Fig. S1A). All training trials and PTs were recorded using EthoVision XT 16 (Noldus) software with colored CCD. The number of target hole visits (correct visits), total errors, total movement distance, velocity, primary latency time, and path tracking were recorded simultaneously for each trial. Collected data were replotted and analyzed using GraphPad Prism 8.0 software.

### Intrahippocampal BDNF knockdown

Vectors and viruses were obtained from the National RNAi Core Factory (Institute of Molecular Biology, Academia Sinica, Taiwan). The target sequence of *Bdnf* shRNA is 5′- TGAGCGTGTGTGACAGTATTA-3′. The lentiviral vector (LV) was bilaterally infused 1 × 10^8^ –1 × 10^9^ particles/ml into the dorsal hippocampus (anteroposterior, − 2.0 mm; mediolateral, ± 1.0 mm; dorsoventral, − 1.8 mm) of anesthetized mice at a rate of 0.1 μl/min by using a microsyringe pump (KDS-101, Kd Scientific). After a 7-day rest period, mice were subjected to behavioral tests. We examined the efficiency of BDNF knockdown by performing western blot analysis after 4 weeks.

### Intracellular Ca2+ measurement

HT-22 cells were seeded in a 35-mm glass bottom dish (1.5 × 10^4^ cells) for 1 day. Cells were incubated for 30 min in the culture medium with Fura-2 AM (2 μM). Subsequently, to examine Ca^2+^ release in the endoplasmic reticulum (ER), cells were washed and placed in a Ca^2+^-free buffer (145 mM NaCl, 5 mM KC1, 10 mM glucose, 1 mM MgCl_2_, and 5 mM HEPES). To examine Ca^2+^ influx, cells were washed and placed in 1.5 mM Ca^2+^ buffer (145 mM NaCl, 5 mM KC1, 10 mM glucose, 1 mM MgCl_2_, 5 mM HEPES, and 1.5 mM CaCl_2_). DMSO and RJW-58 were added to stimulate Ca^2+^ response. In order to identify which calcium channels contribute to the RJW-58 induced Ca^2+^ influx, the HT-22 cells were pre-treated with 10 μM MK-801 (NMDA receptor antagonist) or 10 μM IEM-1754 2HBr (AMPA/kainate receptor blockers, termed as IEM) for 30 min before 5 μM RJW-58 stimulated, and changes in the intracellular calcium ([Ca^2+^]_i_) were recorded. Variations in [Ca^2+^]_i_ were measured and monitored for 15 min at room temperature by using the Fura-2 fluorescence method. To measure the [Ca^2+^]_i_ concentration, cells were exposed to Fura-2, which was excited at wavelengths of 340 and 380 nm. The emitted fluorescence intensity was recorded and analyzed at 510 nm. The [Ca^2+^]_i_ concentration was analyzed using HCImage software. The area under the curve (AUC) of calcium flux was calculated after stimulation by RJW-58 and or DMSO, with background subtraction.

### 5′-Bromo-2′-deoxyuridine injection and immunofluorescence staining of sectioned murine brains

5′-Bromo-2′-deoxyuridine (BrdU) was prepared in warm PBS (pH = 7.4) to obtain a stock solution of 40 mg/ml. BrdU solution was administered (0.2 mg/g body weight) intraperitoneally to mice twice daily at a 4-h interval at indicated time points. Mice were then sacrificed by deeply anesthetizing them with isoflurane. Perfusion was performed by transcardially injecting cold PBS and 10% formalin solution after 24 h when the last doses of RJW-58 and BrdU were administered. Brain tissues were carefully isolated and fixed in 4% paraformaldehyde overnight and then embedded in paraffin. Brain tissues were serially sectioned to a thickness of 5 μm by using a microtome (Leica Biosystems). To detect BrdU-incorporated cells and neural precursor cells after RJW-58 administration, the sectioned slides were deparaffinized by immersion in gradient ethanol solutions. Antigen retrieval was performed using sodium citrate (pH = 6.0) at 95°C–105°C, followed by several washes with PBS–Tween 20 (PBST). The slides were probed with primary antibodies for overnight at 4ºC. Subsequently, appropriate secondary antibodies were conjugated to target molecules. All the antibodies that utilized in this study were listed at Table S1. For analyzing the BrdU-incorporated cells in hippocampus, 6 sections were stained and photographed per mouse. The slides were counterstained with 4',6-diamidino-2-phenylindole (DAPI) before mounting. All brain sections were photographed using a Leica Stellaris 8 confocal image system.

### Image analysis

We assessed the expression of 5-bromo-2'-deoxyuridine (BrdU) in different anatomical sites of the hippocampus tissue. H&E staining was conducted first, hippocampal sections displaying patterns near dorsal side of hippocampus were selected for subsequent BrdU staining, the displaying patterns of dentate gyrus and CA1 regions referred to the previous study [34]. Analysis of all BrdU-incorporated cells and their co-localization with indicated neurogenesis markers was analyzed by using the Slide-based scanning and analysis system (TissueFax system).

### Western blot

Cells and murine hippocampal tissues were carefully isolated from the culture dish and brains, respectively. The isolated cells and hippocampal tissues were lysed with a lysis buffer containing RIPA lysis buffer and a 0.1% phosphatase and protease cocktail. Lysates were additionally homogenized in an ultrasonic homogenizer for 20 s (Model 150 V/T, BioLogics Inc.) prior to centrifugation at 13,000 × *g* for 30 min at 4ºC. Supernatants were preserved, and proteins were separated through sodium dodecyl sulfate–polyacrylamide gel electrophoresis (SDS-PAGE). The separated proteins were transferred to polyvinylidene fluoride (PVDF) membranes (Merck Millipore). The membranes were washed with TBS–Tween 20 (TBST) several times and blocked with 2.5% BSA. Primary antibodies (Table S1) were hybridized to the PVDF membranes overnight at 4ºC. Subsequently, the hybridized membranes were washed three times with PBST and further conjugated with anti-rabbit and anti-mouse horseradish peroxidase (HRP)-conjugated IgG secondary antibodies for 1 h at room temperature. Immunoblot images were visualized using the ECL Western blotting detection reagents (Perkin Elmer) and captured using a Super RX-N medical X-ray film (Fujifilm).

### Nuclear/cytosol protein extraction for SDS-PAGE

Intranuclear or cytosolic proteins were separated as described previously [18]. Briefly, cells were scraped from the culture dish and resuspended in buffer A (pH = 7.9) composed of 10 mM HEPES, 1.5 mM MgCl_2_, 10 mM KCl, 0.5 mM DTT, 0.5% NP-40, 1 mM PMSF, 1mM Na_3_VO_4_, and 0.1% protease inhibitor cocktail. Cell pellets were centrifuged at 3000 rpm for 10 min at 4ºC. The supernatant was collected as the cytosolic fraction of total proteins. Then, cell pellets were washed six to ten times by resuspending the pellets in a wash buffer (buffer A without NP-40) for centrifugation at 13,200 rpm for 5 s, and the supernatant was discarded. Cell pellets were immersed in buffer B (pH = 7.9) containing 5 mM HEPES, 1.5 mM MgCl_2_, 0.2 mM EDTA, 0.5 mM DTT, 26% glycerol, 1 mM PMSF, 1 mM Na_3_VO_4_, 0.1% protease inhibitor cocktail, and 300 mM NaCl for sonication. Then, the cell pellets were centrifuged at 13,200 rpm for 15 min at 4ºC. The supernatant was preserved as the nuclear fraction of total proteins. Later, 20 mg of both the nuclear fraction and cytosolic proteins were subjected to SDS–PAGE after quantification by using a BCA protein assay kit (Thermo Fisher). Separated proteins were transferred to PVDF membranes and treated with primary antibodies (Table S1). Subsequently, the corresponding secondary antibodies were added. The blots were visualized and captured as described in the Western blot subsection.

### Gene expression and real-time quantitative PCR

The total RNA of cultured HT-22 cells was extracted using TRIzol reagent (Thermo Fisher). The extracted RNA was measured using Nanodrop software at absorbance wavelengths ranging from 260 to 280 nm. Complementary DNA (cDNA) was synthesized using the SuperScript III first-strand synthesis system (Thermo Fisher). The mRNA transcription level of *Bdnf* was examined using the StepOnePlus real-time PCR system (Applied Biosystems). The primer sequences are listed in Table S2.

### Chromatin immunoprecipitation (ChIP)

DMSO- and RJW-58-treated HT-22 cells were fixed in 37% formaldehyde for 10 min at 37ºC. The fixed cells were harvested and fragmented to obtain chromatin-DNA through a series of lysis and sonication processes performed on ice. The chromatin-DNA was purified using a ChIP assay kit (Merck Millipore). The purified chromatin-DNA was immunoprecipitated using an anti-olfactory receptor transcription factor (OLF-1) and an IgG control. The binding of OLF-1 to the *Bdnf* promoter was probed using *Bdnf* primers (Table S2).

### Immunoprecipitation (IP)

DMSO- and RJW-58-treated HT-22 cells were lysed by using IP lysis buffer containing 0.5% NP-40, 10% glycerol, 1 mM PMSF, 1mM Na_3_VO_4_, and 0.1% protease inhibitor cocktail in PBS. Later, 5 μg of IgG isotype control and 100 μl of protein A/G sepharose beads were incubated at 4°C for 30 min. Protein lysates were mixed with the beads and incubated overnight at 4°C to pre-adsorb the non-specific binding proteins. The beads were removed and preserved the lysates. Subsequently, 5 μg of anti-OLF-1 antibody or IgG isotype control and 100 μl of protein A/G sepharose beads were incubated at 4°C for 30 min. 1 mg of pre-adsorbed protein lysates were mixed with the beads and incubated overnight at 4°C. The mixture was washed thrice with wash buffer containing 0.5% NP-40 and 0.1% Triton X-100 in PBS to remove non-binding and free proteins and the supernatant was fully discarded. The beads containing captured proteins were then subjected to SDS-PAGE and immunoblotting with appropriate antibodies.

### Promoter assay

Three reporter plasmids, namely OLF1.1-BDNF-pNL2.1 (201 bp), OLF1.2-BDNF-pNL2.1(203 bp), and OLF1.3-BDNF-pNL2.1(202 bp), were generated and purchased from Promega. The concentrations of these reporter plasmids were about 1 μg/μl. The primer sequences are listed in Table S2. 4 μl of reporter plasmids was added into 5 × 10^4^ HT-22 cells by using Lipofectamine 2000 (Thermo Fisher) for 24 h, followed by treatment with 2.5 M RJW-58 for additional 24 h. The cells were assayed using a FlexStation 3 benchtop multimode microplate reader (Molecular Devices) with a 0.5-s integration time to measure luciferase activity.

### Statistical analyses

All in vitro measurements were compiled from at least three independent experiments and are presented as the mean ± standard deviation (SD). Animal behavior tests were performed with a minimum of five mice per group. The results are expressed as the mean ± standard error of the mean (SEM). All data where appropriate were analyzed by using nonparametric ANOVA Kruskal–Wallis test and or Mann–Whitney U test (Y maze, LTP, western blot band analyses), one-way ANOVA (cell, qPCR) and two-way ANOVA with drug dosages or genotypes, and probe trials as the independent variables (Barnes Maze). Where ANOVA revealed a main effect significant between factors, followed by a post hoc comparison test. All tests were performed using GraphPad Prism 8 software. Differences were considered significant if *p* values were < *0.05*.

### Reagents and antibodies

All reagents and antibodies that were utilized in the current study are listed in Tables S1, S2, and S3.

### Data availability

The authors confirmed that the data supporting the current study are available within the article and supplementary material. The raw data that support the findings of current research are also available from the corresponding author, upon reasonable request.

## Results

### Inhibition of CTSS enzymatic activity enhances cognitive functions in animal models

To investigate the effect of targeting CTSS on cognitive functions in vivo, we administered a selective CTSS inhibitor, RJW-58, to wildtype mice (*Ctss*^+*/*+^) for inhibiting the enzymatic activity of CTSS. Next, two Y-maze tests, namely the Y-maze and alternative tests, were performed to evaluate short-term learning and spatial memory, respectively. The timeline of RJW-58 administration and behavioral tests are presented as Fig. 1A. The two Y-maze tests were performed immediately after the administering of RJW-58 for 7 consecutive days at doses ranging from 2.5 to 15 mg/kg. Our data revealed that RJW-58-treated mice spent a longer time in the novel arm especially in 15 mg/kg injection group (*p* < *0.001*, Dunn’s test, Fig. 1B) and exhibited higher alternation rates in 7.5mg/kg (*p* = *0.0058*) and 15mg/kg groups (*p* = *0.0021*, Dunn’s test, Fig. 1C), suggesting that RJW-58 treatment improved learning and spatial memory. Similar results were obtained in CTSS knockout mice (*p* < *0.001*, Mann–Whitney U test, *Ctss*^−/−^ C57BL/6J-Narl), indicating that CTSS plays a pivotal role in regulating working and spatial memory (Fig. 1D, E).

We subjected RJW-58-treated mice and *Ctss*^−/−^ mice to the BM test. We conducted several PTs at indicated timepoints to evaluate long-term learning and spatial memory (Fig. S1A, B and Fig. 1F). To assess learning memory, we administered RJW-58 for 7 consecutive days before training trials and then conducted two PTs at indicated timepoints (Fig. S1B). The learning efficiency of mice was evaluated by calculating the mean latency time required by each mouse to enter the escape box under the target hole during each training trial. Although the mean latency time did not significantly differ between RJW-58 injected mice and the mock group (Two-way ANOVA, *p* > 0.05 in RJW-58 doses; Fig. S1C), *Ctss*^*−/−*^ mice exhibited better learning efficiency than did *Ctss*^+*/*+^ mice (Two-way ANOVA, *p* = *0.0367* in genotypes, *N* = 5, Fig. S1E). This finding suggests that targeting CTSS improves learning efficiency. In addition, *Ctss*^−/−^ mice exhibited significantly improved mean latency time especially at training day 2 (*p* = 0.0147, Sidak’s post hoc test, *N* = 5, Fig. S1E) and significantly improvement in correct visits (Two-way ANOVA, *p* = *0.0214* in genotypes, *N* = 5) particularly at PT-1 (*Ctss*^−/−^, 23.47% ± 3.34%; *Ctss*^+*/*+^,15.77% ± 1.43%, *N* = 5, Mann–Whitney test, *p* = 0.0079, Fig, S1F). To examine long-term spatial memory, we performed a 4-day consecutive training trial and a PT at the acquisition phase prior to a 7-day consecutive dosing of RJW-58, and all mice underwent four PTs every other week. The results indicate significant improvement in primary latency time (Two-way ANOVA, *p* < *0.001, F*_*(3,107)*_ = *9.828* in RJW-58 dose and *p* < *0.001, F*_*(1.755, 62.58)*_ = *17.53* in PTs, *N* = 10/group, the interaction *p* = *0.033* between RJW-58 and PTs) at PT-3 and PT-4 (Fig. 1G), and the percentage of correct visits (Two-way ANOVA, *p* = *0.0002, F*_*(3,36)*_ = *8.537* in RJW-58 dose, and *p* < *0.001, F*_*(2.361, 70.84)*_ = *14.66* in PTs, *N* = 10/group, the interaction *p* = *0.425* between RJW-58 and PTs), at PT-2, PT-3, and PT-4 (Fig. 1H) in RJW-58-treated mice than in the mock group. However, no dose-dependent effect was noted (*N* = 10 mice / group, Fig. 1G, H). Consistently, *Ctss*^−/−^ mice exhibited better performance in terms of both primary latency time (Two-way ANOVA, *p* = *0.0137, F*_*(1,18)*_ = *15.34* in genotype and *p* < *0.001, F* = *30.74* in PTs, *N* = 10/group, the interaction *p* = *0.0014* between genotype and PTs) and the percentage of correct visits (Two-way ANOVA, *p* < *0.001, F*_*(1,18)*_ = *35.46* in genotype and *p* = *0.0002, F*_*(2.332, 30.32)*_ = *10.29* in PTs, *N* = 10/group, the interaction *p* = *0.0014* between genotype and PTs) than did *Ctss*^+/+^ mice in the BM test (*N* = 10 mice, Fig. 1I, J). The path distribution analysis revealed that RJW-58-treated mice had longer target hole contact time than did control mice (Fig. S2C-S2F) at PT-2 (day 19 after RJW-58 injection). Taken together, these results indicate that targeting CTSS can enhance cognitive function in animal models.

### Inhibition of CTSS enhances postsynaptic plasticity and complexity

**Fig. 2:**
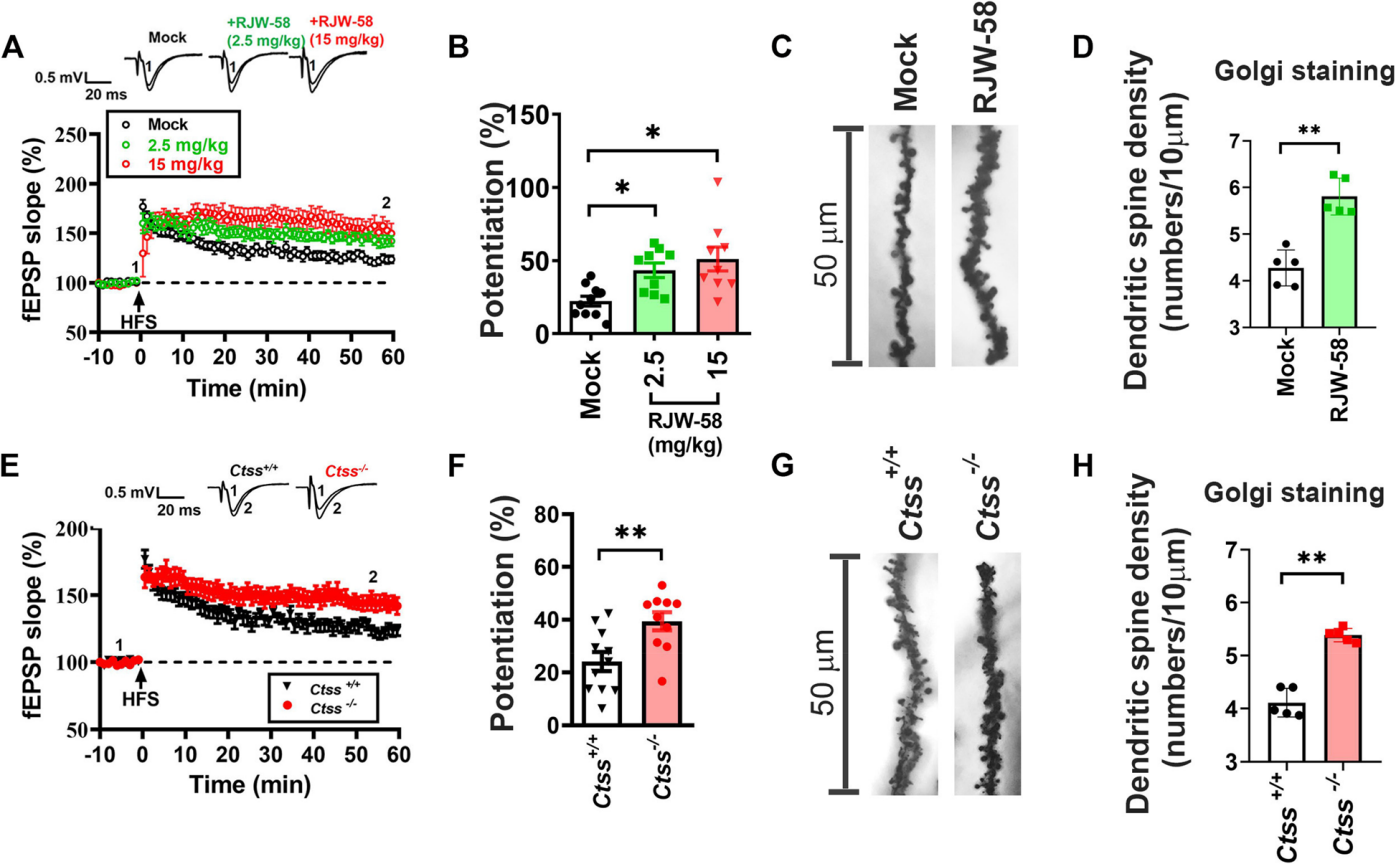
Suppression of CTSS promoted synaptic strength in the CA1 region of the hippocampus. **A**,** E** The fEPSP slope before and after LTP (two trains of 1-s high-frequency stimulation [HFS] at 20-s intervals) induction in hippocampal slices (*n* = 3 slices/mouse) obtained from mice receiving different doses of RJW-58 (*N* = 10 mice/group) (**A**) and *Ctss*^+*/*+^ and *Ctss*^−/−^ mice (*N* = 10) (**E**). **B**, **F** Analyses of the LTP level in hippocampal slices obtained from RJW-58-treated mice (**B**) and *Ctss*^+*/*+^ and *Ctss*^−/−^ mice (F, *p* = *0.0048*). **C**, **G** Representative images of the Golgi staining of hippocampal neurons isolated from control and RJW-58-treated mice (**C**) and from *Ctss*^+*/*+^ and *Ctss*^−/−^ mice (**G**). (Scale bar = 50 μm) (**D**, **H**) Dendric spine density analyses in control and RJW-58 (2.5 mg/kg)-treated mice (*N* = 10) (**D**) and in *Ctss*^+*/*+^ and *Ctss*^−/−^ mice (*N* = 10) (**H**). Bar charts indicate the mean ± SEM. Asterisks indicate significant differences, Dunn’s multiple comparisons *post-hoc* test in B; Mann–Whitney test in C, D, and E; *p < 0.05, **p < 0.01 vs the control group in B and D and the *Ctss*^+*/*+^ group in F and H

On the basis of the findings of neurological behavior models, we propose that the inhibition of CTSS would increase postsynaptic activity, which, in turn, would enhance cognitive functions. Thus, we examined LTP, a classic method used to evaluate synaptic activity in the hippocampal CA1 region. LTP was induced 24 h after the last dose of RJW-58 by using two trains of 1-s high-frequency stimulation (100 Hz) with 20-s intervals (Fig. 2A, E) to stimulate the CA1 region of the hippocampus. Our data revealed that the LTP level was enhanced in RJW-58-treated hippocampal slices compared with control slices. The LTP level increased in high RJW-58 groups (Mock, 24.04% ± 3.57%; 2.5 mg/kg, 44.26% ± 4.58%; 15 mg/kg, 48.67% ± 8.32%; Kruskal Wallis ANOVA test, *p* = *0.0098*, *N* = 9–10 mice/group, Fig. 2B) but reached plateau around 2.5mg/kg. Similarly, the LTP level in *Ctss*^−/−^ mice hippocampal slices was significantly higher than that in *Ctss*^+*/*+^ wildtype mice (*Ctss*^−/−^ mice, 39.37 ± 3.43%; *Ctss*^+*/*+^ mice, 22.21% ± 3.39%, *p* = *0.0021*, Mann–Whitney test, *N* = 10/group, Fig. 2E, F). The findings indicate that the inhibition of CTSS promoted the enhancement of LTP in the murine hippocampal CA1 region, but the higher RJW-58 dosage groups displayed limited enhancement. Therefore, we only choose low RJW-58 injection group (2.5 mg/kg) for later dendritic spine analysis. The dendritic spine density of the hippocampal CA1 region was examined through Golgi staining to investigate the effects of CTSS on synaptic complexity. Golgi staining in the CA1 region revealed significantly higher dendritic spine density in RJW-58 treated mice than in control mice (*p* < *0.001*, unpaired two-tailed *t* test, *N* = 5 mice/group, Fig. 2C, D). Similar results were observed in the hippocampal neurons of *Ctss*^−/−^ mice compared with those of *Ctss*^+*/*+^ mice (*p* < 0.001, unpaired two-tailed *t* test, *N* = 5 mice/group, Fig. 2G, H).

###  Inhibition of CTSS increases Ca ^2+^ influx

**Fig. 3:**
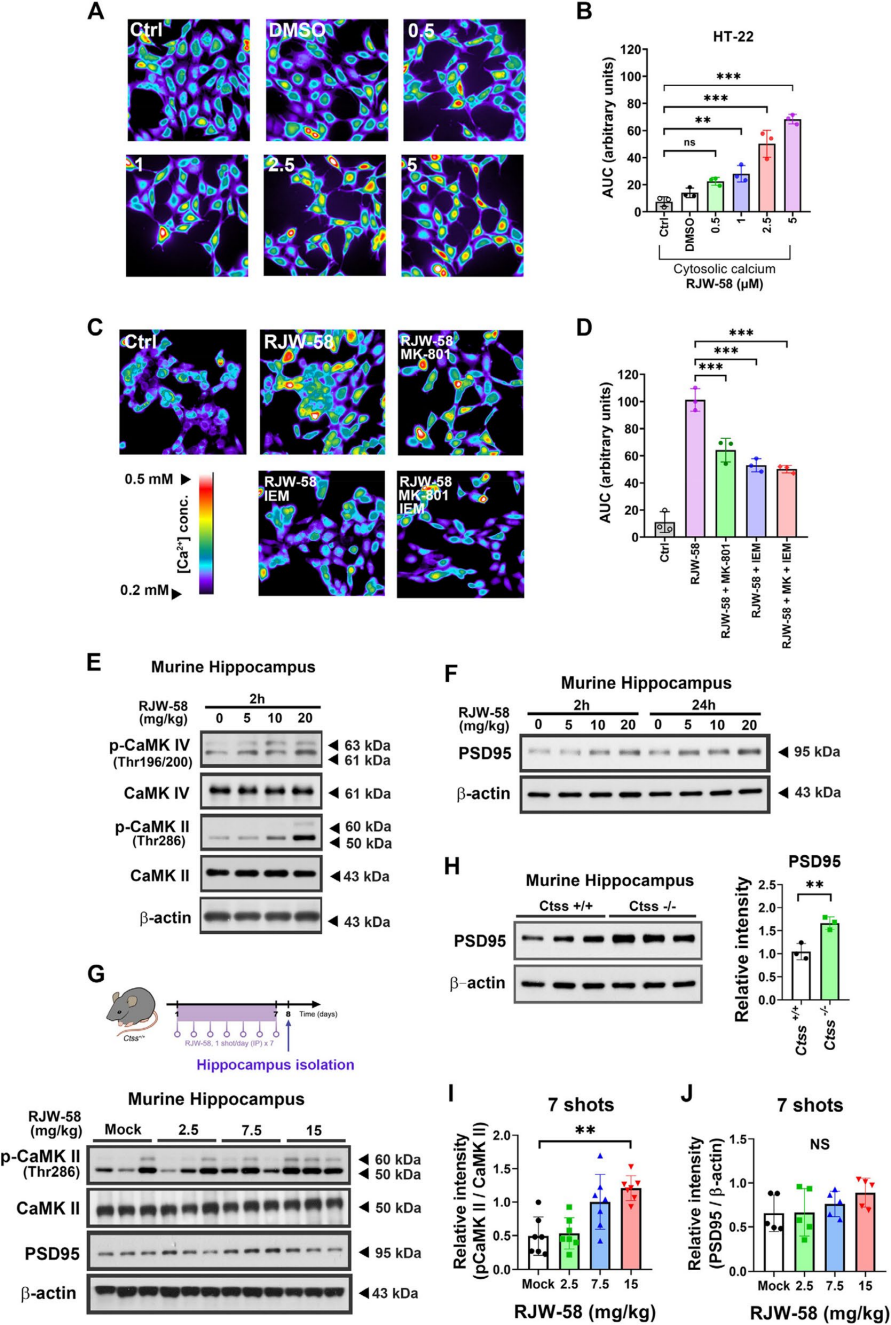
Enhancement of postsynaptic activity is attributed to the augmentation of Ca^2+^ influx in response to the inhibition of CTSS. Fura-2 ratiometric Ca^2+^ imaging of (**A**) HT-22 cells following RJW-58 stimulation (0 to 5 μM) and (**C**) calcium channel blockers. The color scale depicts at (**C**) representing the intracellular calcium concentration within the cytoplasm with relative color coding ranging from blue (low [ Ca^2+^]_i_) to white (high [ Ca2^+^]_i_). Analyses of [Ca^2+^]_i_ fluctuation after (**B**) RJW-58 and (**D**) calcium channel blockers stimulation by measuring the area under curves (AUC) (*N* = 3 independent batches / group). **E** RJW-58 facilitated the phosphorylation of calmodulin kinase II (CaMK II) and CaMK IV in a dose-dependent manner in murine hippocampal tissues after 7-day consecutive treatment with RJW-58. **F** Western blot analysis of PSD95 in murine hippocampal tissues. The mice received a single injection of RJW-58 at concentrations ranging from 0 to 20 mg/kg, and hippocampal tissues were collected at 2 and 24 h after RJW-58 treatment, respectively. **G** Schematic diagram and representative blot of p-CaMKII, CaMK II and PSD95 in hippocampal tissues isolated from *Ctss*^+*/*+^ mice that received a 7-days consecutive injection of RJW-58 at concentration ranging from 0 to 15 mg/kg, the relative band intensity shown as (**I**, **J**). **H** Representative blot of PSD95 expression in hippocampal tissues isolated from 12-week-old *Ctss*^+*/*+^ and *Ctss*^−/−^ mice (*N* = 3 mice/genotype). Bar charts in B and D indicates mean ± SD; in H, I and J indicate the mean ± SEM. Tukey’s test in B; Mann–Whitney test in F; Dunn’s *post-hoc* test in G and H; Asterisks indicate significant differences, **p < *0.01* versus the control group in B, versus the *Ctss*^+/+^ group in F, versus the Mock group in G and H

Ca^2+^ plays a crucial role in the regulation of synaptic plasticity [35]. Our studies have demonstrated that the expression level of CTSS plays a role in the regulation of intracellular Ca^2+^ homeostasis [18, 21]. We determined whether RJW-58 can directly affect the intracellular Ca^2+^ dynamic of neurons by monitoring the [Ca^2+^]_i_ concentration in mouse hippocampal neuronal cells (HT-22) after RJW-58 treatment. Ratiometric imaging of Ca^2+^ revealed that Fura-2 fluorescence intensity increased following RJW-58 treatment (Fig. 3A, S3A), indicating that RJW-58 treatment significantly increased the [Ca^2+^]_i_ concentration in a dose-dependent manner (AUC [ Ca^2+^]_i_ = 7.524 ± 2.841 Arbitrary Units (AU) in control; 14.117 ± 2.875 AU in DMSO; 22.497 ± 2.32 AU in 0.5 μM RJW-58; 28.13 ± 4.94 AU in 1 μM RJW-58; 50.257 ± 8.109 in 2.5 μM RJW-58; 68.353 ± 2.87 in 5 μM RJW-58, One-way ANOVA, *F*_*(5,12)*_ = *53.54*, *p* < *0.001*, *N* = 3/group, Fig. 3A, B, and S3A). In the other hand, calcium release from the endoplasmic reticulum (ER) did not significantly reveal difference (*N* = 3/group, Fig. S3B, C). Intriguingly, the delayed onset of the Ca^2+^ peak in the HT-22 cells treated with higher doses of RJW-58 (Fig. S3A) could potentially be attributed to extracellular Ca^2+^-induced intracellular Ca^2+^ release from the ER [36]. To further elucidate which types of calcium channels interact with RJW-58, we utilized the NMDA receptor antagonist MK-801 and the AMPA calcium channel blocker IEM 1754 2HBr (referred to as IEM) in the following analysis. After pre-treatment with these calcium channel blockers for 30 min, we observed that the RJW-58 mediated calcium influx was partially suppressed by both MK-801 and IEM. (AUC [ Ca^2+^]_i_ = 11.01 ± 6.24 AU in control; 101.157 ± 6.828 AU in 5 μM RJW-58; 64.1 ± 7.145 AU in 5 μM RJW-58 + 10 μM MK-801; 53.01 ± 3.94 AU in 5 μM RJW-58 + 10 μM IEM; 50.133 ± 2.2 AU in 5 μM RJW-58 + 10 μM MK-801 + 10 μM IEM, One-way ANOVA, *F*_*(4,10)*_ = *66.46*, *p* < *0.001*, *N* = 3/group, Fig. 3C, D). This suggests that RJW-58 mediates calcium influx by interacting with NMDA and AMPA receptors. However, the effect of RJW-58 was not fully eliminated after treatment with dual inhibitors, suggesting the possibility of calcium flux mediated by RJW-58 through other types of calcium channels. Taken together, although our results suggest that RJW-58 treatment predominantly promotes extracellular Ca^2+^ influx rather than Ca^2+^ release from intracellular stores such as the ER, we still cannot fully explain the alterations of Ca^2+^ channel activity on the cell membrane once CTSS is inhibited. Further study is warranted to unveil the underlying mechanisms of how RJW-58 alters calcium entry through the calcium channels.

Elevated Ca^2+^ influx drives downstream signaling pathways that regulate cascades in the central nervous system (CNS). We examined the levels of Ca^2+^/calmodulin kinases (CaMKs) in hippocampal tissues after RJW-58 treatment. Our data revealed that CaMK II and CaMK IV in murine hippocampal tissues were phosphorylated within 2 h in a dose-dependent manner after a single injection of RJW-58 (*N* = 5 mice/group, Fig. 3E, S3D-E). Later, we analyzed the expression level of postsynaptic density protein 95 (PSD95), a critical protein that can form a complex with CaMK II to regulate synapse formation in murine hippocampal tissues [37]. Our findings revealed that the expression level of PSD95 increased both 2 and 24 h after RJW-58 injection in a dose-dependent manner (*N* = 5 mice/group, Fig. 3F, S3F-G). We administered RJW-58 for 7 consecutive days at designated dosages ranging from 0 to 15 mg/kg (Fig. 3G). We observed the phosphorylation of CaMK II in hippocampal lysates in a dose-dependent manner (*N* = 7, Fig. 3G, I). However, repeating dosing of RJW-58 lead to not significantly difference (*p* = *0.176*, *N* = 5, Kruskal–Wallis ANOVA test) on PSD95 expression despite the relative intensity is higher in 15mg/kg RJW-58 injection groups (*p* = *0.1113*, Dunn’s test, Fig. 3G, J). Consistently, the PSD95 level in *Ctss*^−/−^ mouse hippocampal tissues was significantly higher than that in *Ctss*^+*/*+^ mouse hippocampal tissues (*N* = 3, *p* = *0.0087*, unpaired two-tailed *t* test, Fig. 3H). Taken together, our data indicate that suppressing the enzymatic activity of CTSS facilitated Ca^2+^ influx, which then promoted the expression of PSD95, enhancing postsynaptic activity in the murine hippocampus.

### Effect of RJW-58 on neurogenesis

**Fig. 4:**
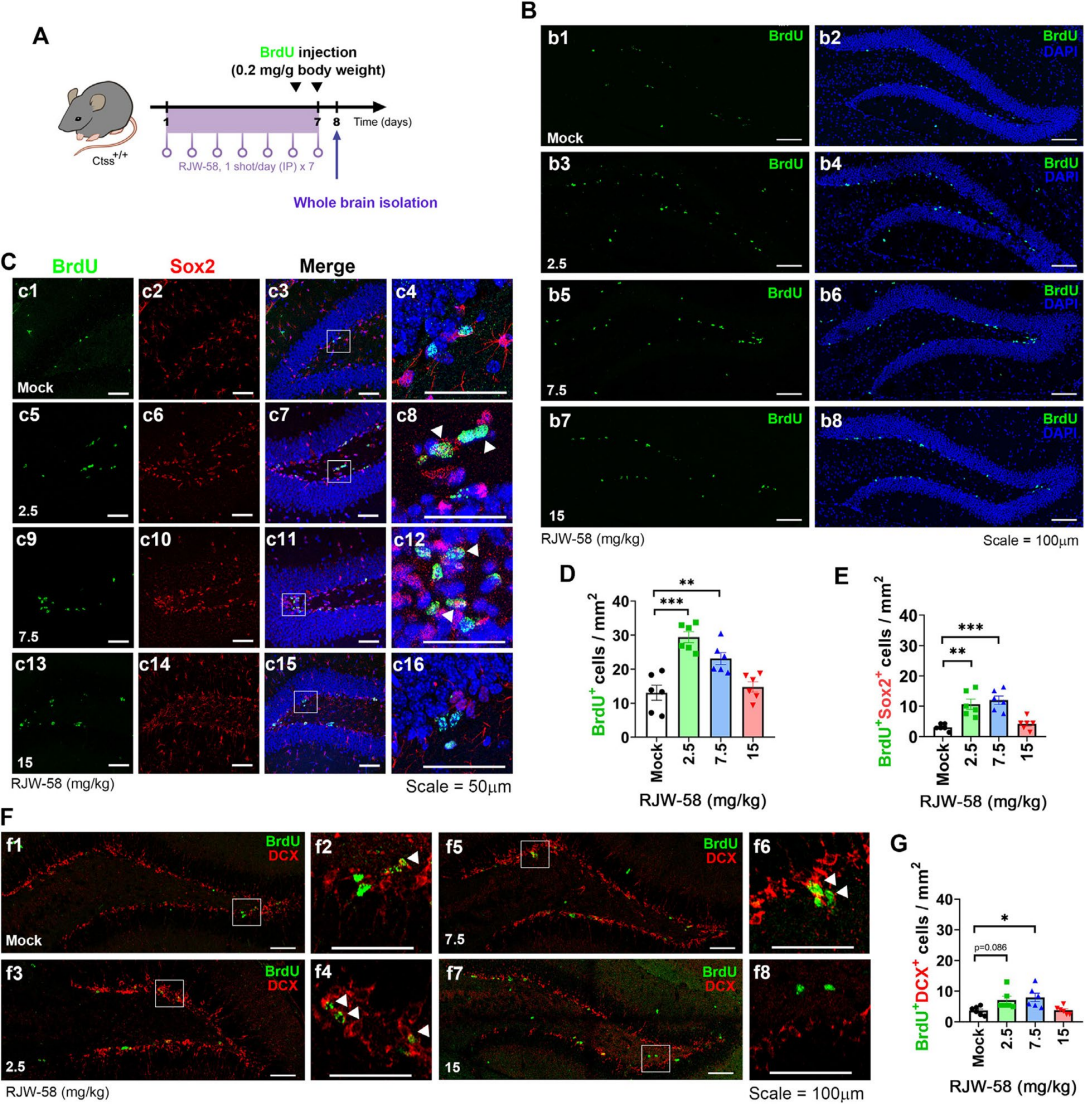
Efficiency of RJW-58 in facilitating the activation of neuronal precursor cells in the dentate gyrus region. **A** Schematic displaying the timeline of RJW-58 dosing and BrdU labeling. **B** Representative images of BrdU-incorporated cells in the dentate gyrus after 7-day consecutive treatment with RJW-58 at designated dosages (numbers labeled at the lower left of panels b1, b3, b5, and b7; BrdU was stained green, and DAPI was stained blue. Scale bar = 200 μm). Representative images of BrdU^+^Sox2^+^ cells (**C**) and BrdU^+^DCX^+^ cells (**F**) in the dentate gyrus region of the hippocampus in different RJW-58 treatment groups. The outlined square frames in merge panels are shown at right panels (BrdU^+^Sox2^+^ cells: c4, c8, c12, and c16; BrdU^+^DCX^+^ cells: f2, f4, f6, and f8) where arrowheads highlight cells expressing colocalized BrdU and targets, indicating the presence of neuronal precursor cells and the progression of neurogenesis in the dentate gyrus (BrdU was stained green, Sox2 and DCX were stained red, and DAPI was stained blue.) **D**, **E **and **G** Quantitative analyses of the number of BrdU-incorporated cells (**D**), BrdU^+^Sox2^+^ cells (**E**) and BrdU^+^DCX^+^ cells (**G**) in the dentate gyrus (*N *= 6 mice / group). 6 hippocampal sections were stained and analyzed per mouse, all BrdU^+^, Sox2^+^, DCX^+^ cells or co-expression cells were normalized to the hippocampus area (mm^2^) to obtain cell numbers per mm^2^. The average hippocampus surface areas were shown as below: Mock, 0.76 ± 0.06 mm^2^; 2.5 mg/kg, 0.79 ± 0.061 mm^2^; 7.5 mg/kg, 0.78 ± 0.184 mm^2^; 15 mg/kg, 0.72 ± 0.14 mm^2^. Bar charts indicate the mean ± SEM. Asterisks indicate significant differences, Dunn’s *post-hoc* test, *p < *0.05*, **p < *0.01*, ***p < *0.001* versus the Mock group in D, E and G. Scale bar had indicated in the figures

To investigate the effect of RJW-58 on neurogenesis, we injected BrdU parallelly to the last two doses of RJW-58. The whole brain was perfused and carefully isolated 24 h after injection (Fig. 4A). Subsequently, BrdU-incorporated (BrdU^+^) cells were probed and quantified through immunofluorescence staining. Quantitative analyses of BrdU^+^ cells revealed the presence of more BrdU^+^ cells in the dentate gyrus (DG) region of the hippocampus in the low-dose groups (Mock, 13.08 ± 2.26 cells/mm^2^; 2.5 mg/kg, 29.42 ± 1.61 cells/mm^2^; 7.5 mg/kg, 23.13 ± 1.77 cells/mm^2^, *n* = 6 sections/mouse, *N* = 6 mice/group) but not the high-dose groups (15 mg/kg, 14.78 ± 1.52 cells/mm^2^; Kruskal–Wallis ANOVA test, *p* < *0.0001,* Fig. 4B, D). The co-staining of Sox2, a predicted marker for residential neuron precursor cells in the brain, indicated the co-localization pattern of BrdU^+^ cells, suggesting the activation of type 2a neuron precursor cells (Fig. 4C). Similarly, more BrdU^+^Sox2^+^ cells were noted in the low-dose groups (Mock, 3.09 ± 0.44 cells/mm^2^; 2.5 mg/kg, 7.12 ± 1.28 cells/mm^2^; 7.5 mg/kg, 7.99 ± 1.37 cells/mm^2^; 15 mg/kg, 3.78 ± 0.53 cells/mm^2^; Kruskal–Wallis ANOVA test, *p* < *0.001,n* = 6 sections/mouse, *N* = 6 mice/group; Fig. 4E). These data demonstrate that RJW-58 evokes quiescent neuron precursor cells in DG but may have a dose range activity. Subsequently, we analyzed the BrdU^+^ and doublecortin (DCX^+^) co-expression cells, which demonstrated the progressive stage marker of the neurogenesis during development [38, 39]. Our data demonstrated that more BrdU^+^DCX^+^ co-expression cells were observed in lower but not high RJW-58 injected groups (Mock, 3.69 ± 0.57 cells/mm^2^; 2.5 mg/kg, 7.12 ± 1.68 cells/mm^2^; 7.5 mg/kg, 11.98 ± 1.41 cells/mm^2^; 15 mg/kg, 4.19 ± 0.8 cells/mm^2^; Kruskal–Wallis ANOVA test, *p* = *0.0091,n* = 6 sections/mouse, *N* = 6 mice/group; Fig. 4F, G). However, we did not find significantly increase in BrdU^+^NeuN^+^ co-expression cells upon all groups (Fig. S4A-C), suggesting that the timeline in our study did not fully cover the whole neurogenesis process, which might spent more than 100 days to differentiate into NeuN^+^ cells from neuron stem/progenitor cells in vivo [40]. Subsequently, we analyzed the stage-specific neurogenesis markers in adult Ctss^−/−^ mice. The results had shown that no significant increase in BrdU^+^, Sox2^+^, DCX^+^, and NeuN^+^ cells in *Ctss*^−/−^ hippocampal tissues (*N* = 3 mice/genotype, Fig. S4D-J), suggesting that long-term alterations of physiological homeostasis attribute to knockout CTSS might lead to the results that is differential from the transient pharmacological blockade of CTSS. The role of CTSS in neurogenesis should be further investigated in the future.

### CTSS knockdown leads to the upregulation of BDNF

**Fig. 5:**
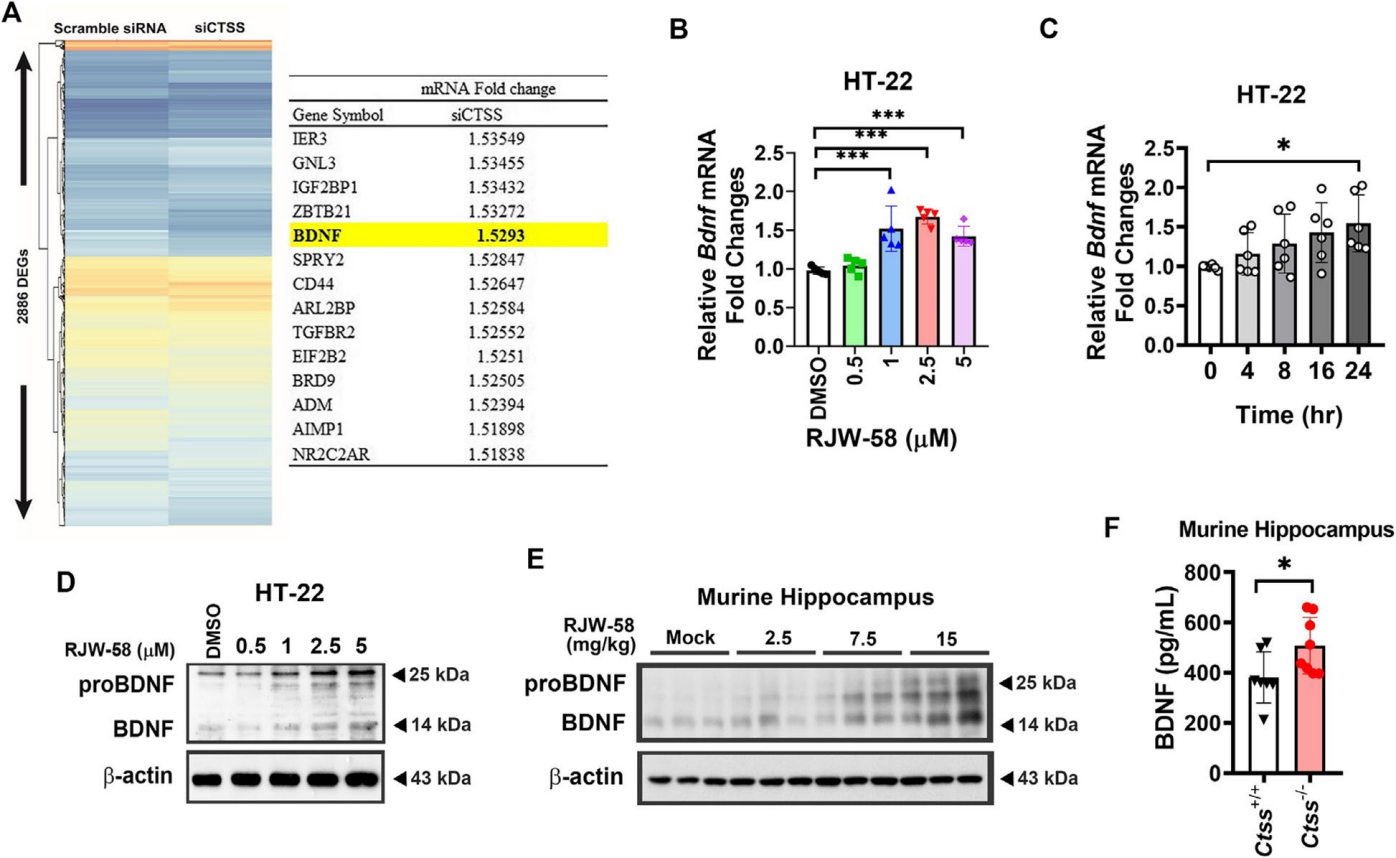
Knockdown of CTSS upregulated BDNF gene transcription and protein expression. **A** Clustering and heat map of the microarray analysis between the scramble siRNA and siCTSS groups of OEC-M1 cancer cells. The table indicates the mRNA fold change in siCTSS compared with scramble siRNA. Transient CTSS knockdown upregulated the mRNA expression of *Bdnf* (mRNA fold change level in the siCTSS group was normalized to that of the scramble siRNA group, fold-change cutoff ≥ 1.5 is shown in the table). **B** RJW-58 treatment enhanced *Bdnf* gene expression in HT-22 cells in a dose-dependent manner (*N* = 5). **C** Upregulation of *Bdnf* expression from 0 to 24 h after RJW-58 (2.5 µM) treatment (*N* = 6 per time window). The representative immunoblotting images of proBDNF and BDNF in (**D**) RJW-58 treated HT-22 cells after 48 h, and (**E**) the murine hippocampal tissues which were received 7-day consecutive RJW-58 dosing. **F** ELISA was used to validate the higher BDNF protein level expressing in the *Ctss*^−/−^ knockout mice hippocampal tissues than in that of *Ctss*^+*/*+^ wildtype mice (*N* = 7). Bar charts indicate the mean ± SD. Asterisks indicate significant differences, Dunnett’s *post-hoc* test in B and C, unpaired two-tailed *t*-test in F, *p < *0.05* versus the DMSO group in B, versus the 0-h group in C, versus *Ctss*^+*/*+^ mice in F

We demonstrated that the transient transfection of siRNA to knockdown CTSS in cancer cell lines led to the upregulation of 14 genes by approximately 1.5-fold (Fig. 5A). Together with our data from neurological behavior tests, we propose that the knockdown of CTSS would remodel the microenvironment, resulting in beneficial alterations in residential cells and tissues. BDNF, a crucial neurotrophic factor in the CNS, was identified (Fig. 5A). To validate the gene expression profiling results, we examined the effect of RJW-58 on BDNF expression in a mouse hippocampal cell line, HT-22. Our data demonstrated that the expression levels of BDNF mRNA increased in a dose- (*N* = 5, One-way ANOVA, *F*_(4,20)_ = *18.87, p* < *0.001*, Fig. 5B) and time-dependent manner (*N* = 6, One-way ANOVA, *F*_(4,45)_ = *3.503, p* = *0.0142*, Fig. 5C). Moreover, BDNF protein level exhibited consistent results (*N* = 5, One-way ANOVA, *F*_(4,20)_ = 12.26, *p* < 0.001, Fig. 5D, S5A, and S5C). Similarly, *Ctss*^−/−^-derived primary cortical neurons exhibited higher pro-BDNF and BDNF levels than *Ctss*^+*/*+^-derived cells (Fig. S5F). RJW-58 treatment increased both pro-BDNF and BDNF expressions in murine hippocampal tissues in a dose-dependent manner (*N* = 6 mice/group, Kruskal–Wallis ANOVA test, *p* = *0.0081*, Fig. 5E, S5B). ELISA analysis revealed that the protein level of BDNF in hippocampal tissues isolated from *Ctss*^−/−^ mice was significantly higher than in those isolated from *Ctss*^+*/*+^ mice (*p* = 0.037, *unpaired two-tailed t test*, *N* = 7 tissues/genotype, Fig. 5F).

### Involvement of OLF-1 in RJW-58-mediated BDNF regulation

**Fig. 6:**
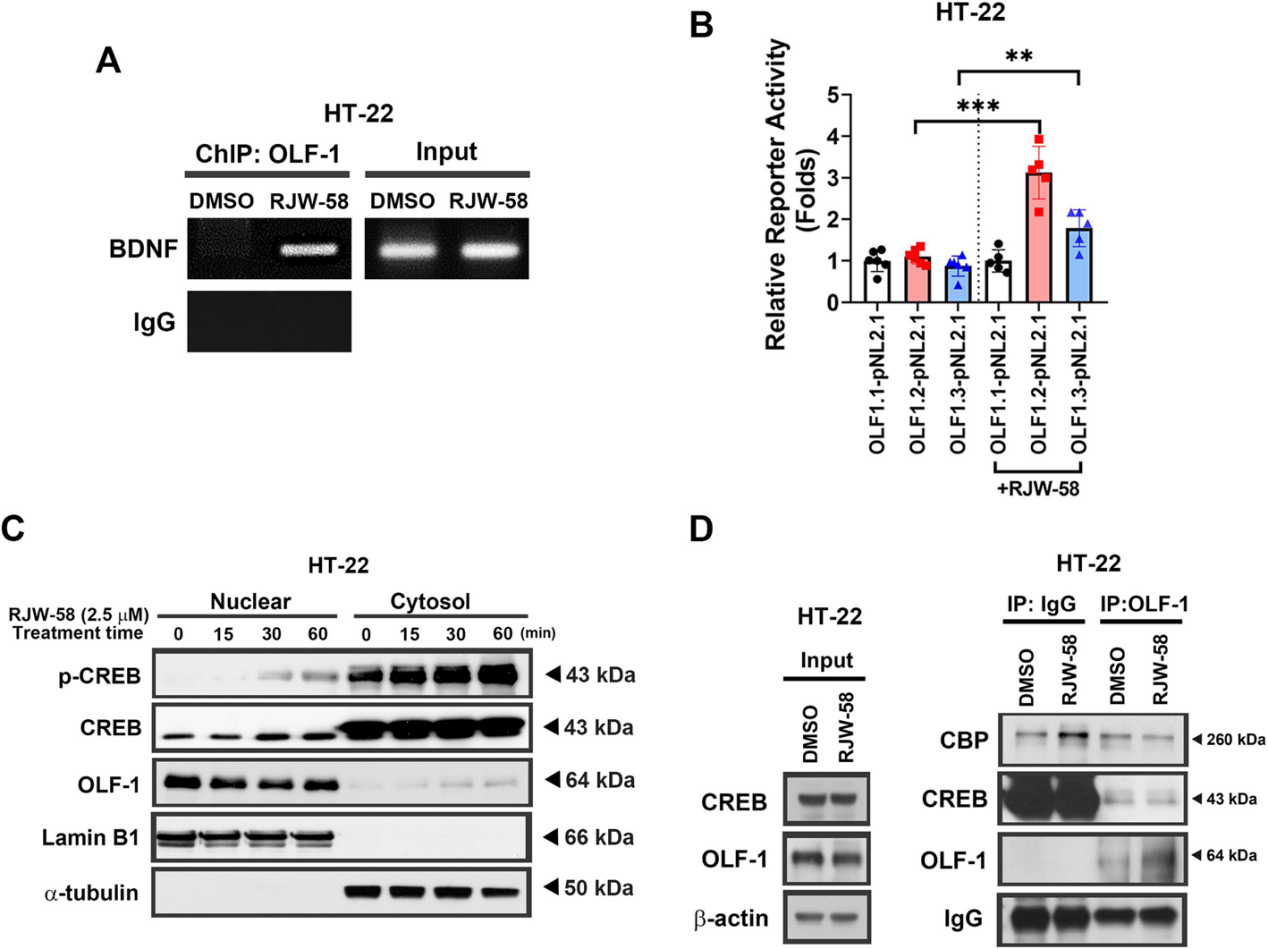
Involvement of OLF-1 in BDNF regulation. **A** The representative images of chromatin immunoprecipitation of RJW-58-treated HT-22 cells indicated OLF-1 binding to the BDNF promoter. **B** Promoter activity assay of *Bdnf* gene in HT-22 cells before and after 24-h treatment with 2.5 μM RJW-58 (*N* = 5–6 / promoter region). **C** Treatment with RJW-58 (2.5 µM) in HT-22 cells promoted the phosphorylation of CREB and translocated it into the nucleus within 30 min; OLF-1 was then translocated into the cytosol. **D** Immunoprecipitation analysis indicated that OLF-1 was released from the CBP/CREB complex following RJW-58 treatment. Bar charts in B indicate the mean ± SD. Asterisks indicate significant differences, unpaired two-tailed *t*-test in B, **p < *0.01*, ***p < *0.001* versus the corresponded promoter region before RJW-58 treatment

Our previous study demonstrated that the inhibition of CTSS resulted in the release of OLF-1 from the CREB-binding protein (CBP) and its binding to the promoter region of *Il10* [18]. We performed the ChIP assay to determine whether OLF-1 can bind to the promoter region of *Bdnf*. Our results indicate that OLF-1 bound to the *Bdnf* promoter when HT-22 cells were treated with RJW-58 (*N* = 3, Fig. 6A). We performed a luciferase functional quantitative assay to investigate the effects of RJW-58 on *Bdnf* promoter activity. Three pairs of primers were designed (Fig. S6A). Compared with the OLF1.1-pNL2.1 vector, RJW-58 led to a threefold increase in BDNF transcriptional activity, especially at the binding site of OLF1.2-pNL2.1 (*N* = 6, unpaired two tailed *t*-test, *p* < *0.001*, Fig. 6B). In addition, our results indicate that RJW-58 treatment increased the level of p-CREB in the nucleus (*N* = 3, One-way ANOVA, *F*_*(3,8)*_ = *17.87*, *p* < *0.001*, Fig. 6C, Fig. S6C), with a concomitant increase in OLF-1 expression in the cytoplasm in a time-dependent manner (*N* = 3, One-way ANOVA, *F*_*(3,8)*_ = *4.031*, *p* = *0.051*, Fig. 6C, Fig. S6B), the statistical analysis revealed significantly between 0 and 60 min (Dunnett’s test, *p* = *0.0294*, Fig. S6B). These results suggest the translocation of OLF-1 from the nucleus to cytoplasm by 2.35 times within 60 min after RJW-58 treatment. Consistent with the finding of our previous study [18], the IP experiment demonstrated that RJW-58 treatment reduced the binding of OLF-1 to the CBP/CREB complex and increased free OLF-1 by 1.54 folds (*N* = 3, unpaired two-tailed *t*-test, *p* = *0.0392*, Fig. 6D, S6D-F) and facilitated the dissociation of CBP from CBP/CREB complex (*N* = 3, unpaired two-tailed *t*-test, *p* = *0.016*, Fig. 6D, S6D-F). Taken together, our data demonstrated that the translocation of OLF-1 from the nucleus to cytoplasm by reducing the binding affinity on CBP/CREB complex. Therefore, dissociated CREB and OLF-1 from OLF-1/CBP/CREB complex are capable to bind onto *Bdnf* promoter regions and activate the *Bdnf* transcription.

### Effects of RJW-58 on the regulation of BDNF/TrkB signaling

**Fig. 7:**
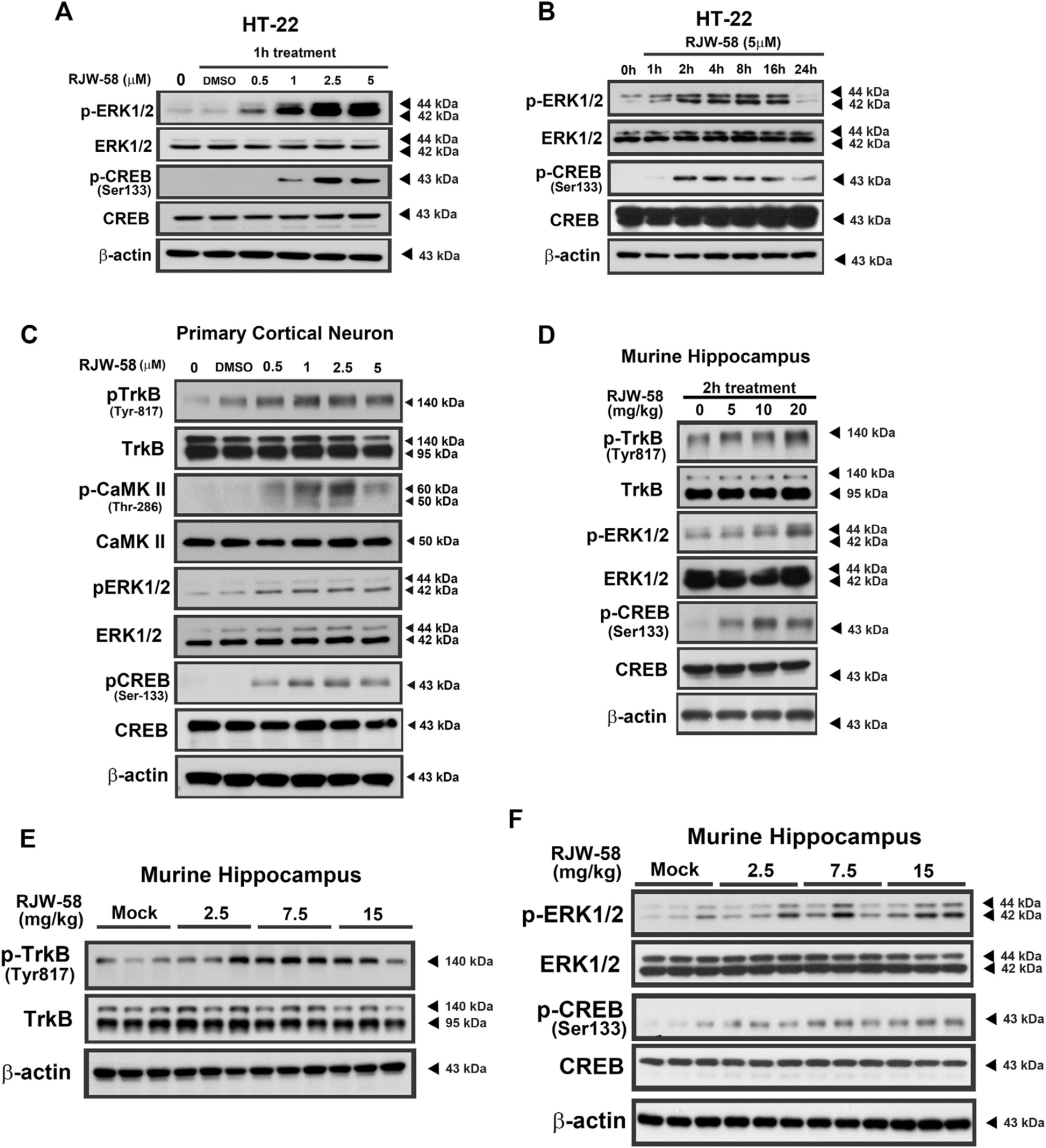
Effects of RJW-58 on regulating BDNF/TrkB signaling. **A**, **B** The representative western blot images of the downstream cascades of BDNF/TrkB signaling after treatment with 0–5 µM RJW-58 for 1 h (**A**), and time course blotting of BDNF/TrkB signaling in HT-22 cells after RJW-58 treatment from 0 to 24 h (**B**). **C** The representative western blot images of cultured primary cortical neurons treated with RJW-58 for 2h to activate BDNF/TrkB signaling through phosphorylating the TrkB at Tyr-817 residues. The phosphorylation of downstream CaMK II, ERK, and CREB revealed a dose-dependent effect. **D** The in vivo effect of RJW-58 on activating BDNF/TrkB signaling through the phosphorylation of Tyr-817 residues on TrkB in murine hippocampal tissues after single dose of RJW-58 administration at concentrations ranging from 0 (Mock) to 20 mg/kg for 2h. **E**, **F** The representative protein blotting images of the murine hippocampus for the downstream BDNF/TrkB axis at the 8th day (**E**) and 7th week (**F**) after a 7-day-consecutive RJW-58 administration at concentrations ranging from 0 (Mock) to 15 mg/kg at day 0. Hippocampal tissues were collected at the eighth day and seventh week, respectively

**Fig. 8:**
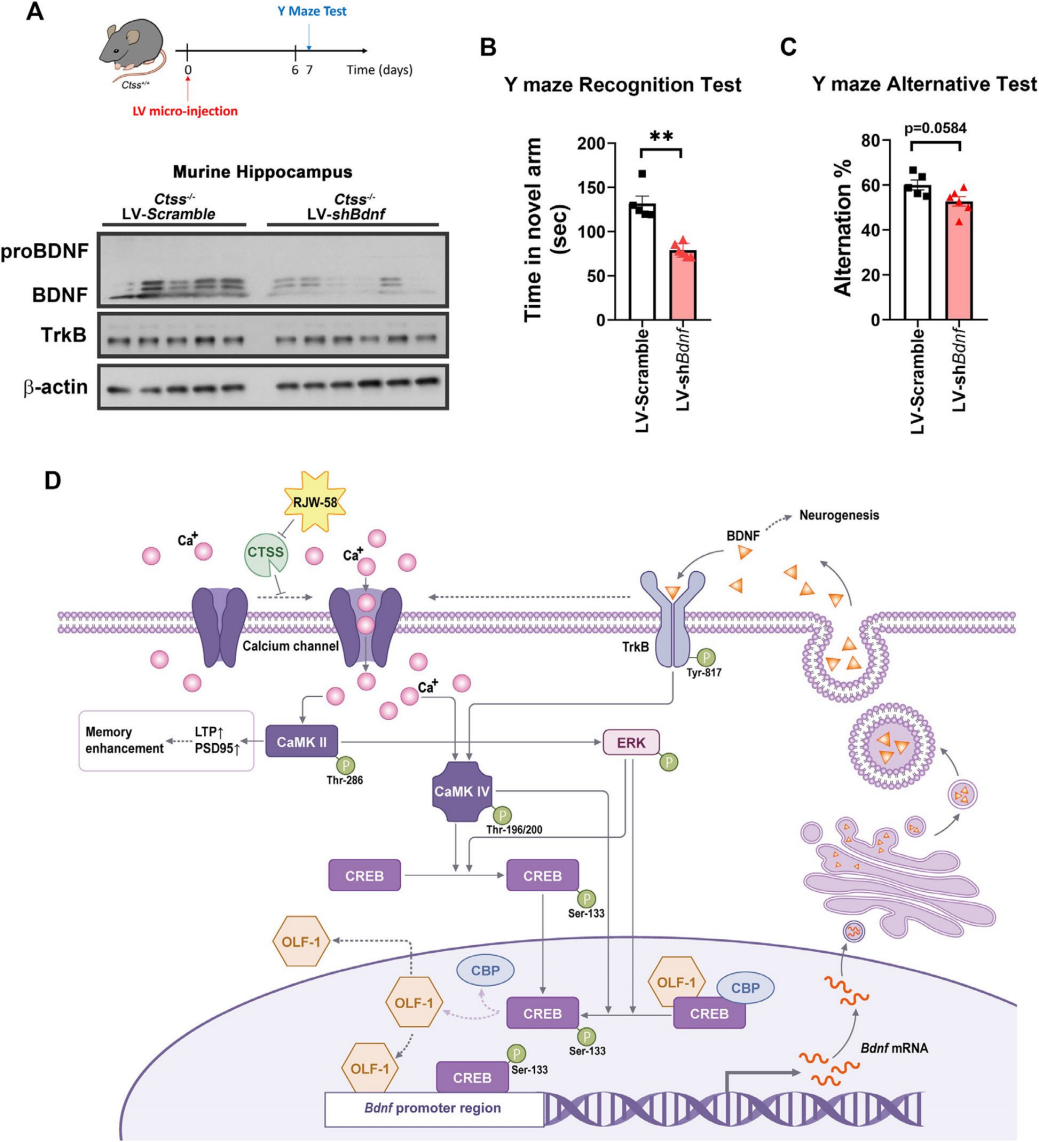
Promoting effect of targeting CTSS on cognitive functions is eliminated by transcriptionally suppressing BDNF. **A** The timeline of LV intrahippocampal injection and behavior tests performed in *Ctss*^−/−^ mice. Western blot analysis of BDNF expression after 4 weeks of LV intrahippocampal injection. Analysis of working memory and spatial memory in the Y-maze recognition (**B**) and alternation test (**C**) when *Ctss*^−/−^ mice were transfected with LV-scramble and LV-sh*Bdnf* (*N* = 5–6 mice/group). **D** Proposed model through which targeting CTSS by RJW-58 triggers BDNF/TrkB-OLF-1 signaling in neurons. Arrows with dashed lines represent speculated pathways, while arrows with solid lines indicate validated pathways in this study. Bar charts in B indicate the mean ± SEM. Asterisks indicate significant differences, Mann–Whitney test, *p < *0.05*, **p < *0.01*, versus the LV-scramble group

To elucidate BDNF downstream signaling pathways, we incubated HT-22 cells in the presence and absence of RJW-58 at different timepoints. Western blot analysis indicated that RJW-58 treatment increased the phosphorylation of ERK (p-ERK) and CREB (p-CREB) but not Akt (Fig. S8C-D) in a time- and dose-dependent manner (*N* = 3, Fig. 7A, B, and S7A-D). Because of the lack of the BDNF receptor TrkB in HT-22 cells, primary cortical neuron cells isolated from the neonatal cortex of *Ctss*^+*/*+^ mice were used to determine the phosphorylated site of TrkB after RJW-58 treatment. Our data demonstrate that RJW-58 treatment increased the phosphorylation of TrkB at Tyr-817 but not Tyr-515 and Tyr-707 (Fig. S8A), p-CaMK II, p-ERK1/2, and p-CREB in a dose-dependent manner (*N* = 3, Fig. 7C, S7E-H). We also analyzed the BDNF/TrkB axis in vivo. *Ctss*^+*/*+^ mice were treated with different doses of RJW-58 in one shot to further investigate the underly mechanism. Western blot analysis of hippocampal tissues confirmed the phosphorylated site of TrkB at Tyr-817 in RJW-58-treated mice (*N* = 4 mice/group, Fig. 7D, S7I). In addition, p-ERK and p-CREB were detected within 2 h after RJW-58 injection, and the intensity of phosphorylation increased in a dose-dependent manner (*N* = 4 mice/group, Fig. 7D, S7J-K). Moreover, *Ctss*^+*/*+^ mice were administered RJW-58 for 7 consecutive shots at designated dosages once a day, and their murine hippocampal tissues were carefully dissected 24 h and 7 weeks after RJW-58 administration. Our results indicate that RJW-58 treatment increased the phosphorylation of TrkB at Tyr-817 (Fig. 7E, S7I) and CaMK II at Thr-286 after 24 h (Fig. 3E, S3E), which represented short-term or immediate signaling triggered by RJW-58. The signal of p-ERK and p-CREB persisted at the seventh week (*N* = 6 mice/group, Fig. 7F), unlike that of p-TrkB at Tyr-817 (Fig. S8B), suggesting that targeting CTSS leads to the long-lasting effect of the OLF1-BDNF/TrkB axis. When hippocampal BDNF expression was reduced in *Ctss*^*−/−*^ mice by using LV-*shBdnf,* the BDNF expression was knocked down, but the TrkB expressed normally (Fig. 8A). We noted a decline in working and spatial memory in behavioral tests compared with *Ctss*^*−/−*^ mice treated with scramble control (*N* = 5–6 mice/group, Fig. 8B-C). These findings suggest that the induction of BDNF expression is required to observe the effect of CTSS inhibition on working and spatial memory.

### Proposed model of suppressing CTSS on regulating BDNF/TrkB-OLF-1 axis

Herein, we propose a model to interpret the modulation of the BDNF/TrkB-OLF-1 axis in the CNS (Fig. 8D). The inhibition of the enzymatic activity of CTSS results in an increase in intracellular Ca^2+^ concentrations and subsequently leads to the phosphorylation of CaMK II and CaMK IV. The phosphorylation of CaMK II further promotes LTP and PSD95, which then enhances postsynaptic plasticity and complexity. Phosphorylated CaMKII triggers the phosphorylation of ERK, which promotes the phosphorylation of CREB. By contrast, the phosphorylation of CaMK IV directly facilitates CREB phosphorylation. Subsequently, phosphorylated CREB translocate from the cytosol into the nucleus, resulting in the dissociation of the intranuclear OLF-1/CREB/CBP protein complex, accompanied by the translocation of OLF-1 from the nucleus to the cytosol. Eventually, OLF-1 is released and binds to the *Bdnf* promoter region, which increases the transcription of BDNF. Later, BDNF is synthesized and released to promote neurogenesis or to bind to TrkB by phosphorylating the Tyr-817 site and form a loop of the OLF-1-BDNF/TrkB signaling pathway. Our data provide a novel strategy and insight for regulating BDNF signaling and postsynaptic plasticity by targeting CTSS.

## Discussion

CTSS plays a major role in the inflammatory processes of various neurodegenerative diseases and aging. The accumulation of CTSS in the CNS might serve as a critical factor for neuroinflammation, contributing to cognitive function impairment [41]. However, the underlying mechanism of CTSS in the CNS remains elusive. This study demonstrates that suppressing CTSS improved working and long-term memory, as observed in the Y-maze and BM tests. Our data indicate that the inhibition of CTSS by RJW-58 induced CaMKII-CREB signaling, increasing BDNF expression, LTP level, and synaptic spine density in the hippocampus. RJW-58 treatment promoted OLF-1 translocation coordinated with CREB, increasing *Bdnf* transcription. Similar results were obtained for *Ctss*^*−/−*^ mice. Understanding the role of CTSS in regulating memory may help to develop an innovative strategy to mitigate cognitive impairment. Our work provides novel insights into targeting CTSS to regulate synaptic plasticity and memory.

The expression level of CTSS was increased in an aged mouse [19]. The aging brain exhibits chronic inflammation and cognitive decline, especially in working memory [42, 43]. In clinical studies, an elevated plasma CTSS level was noted in patients with Alzheimer’s disease [44] and was correlated with increased risks of immobility and mortality in older adults [45, 46]. In addition, animal studies have reported that CTSS in the mouse brain increased with aging [47, 48], and the mature form of CTSS increased in activated astrocytes and microglia in the hippocampus of aged mice [19]. Accumulated evidence suggests a strong association between CTSS and neurodegenerative disease as well as aging. Elevated CTSS has been observed in high-grade astrocytoma [49] and aging [19]. Immunohistochemical analyses have further revealed intensified CTSS distribution in Aβ-positive plaques and τ-positive tangle-bearing neurons in Alzheimer's disease brain sections [17], indicating that elevated CTSS levels might disrupt physiological homeostasis in the microenvironment and contribute to pathological changes as well as cognitive impairment in the CNS. Our study demonstrated that targeting CTSS promotes cognitive function by enhancing synaptic complexity and potentially evoking neurogenesis. This suggests that suppressing CTSS could be a potential therapeutic target to ameliorate cognitive impairment. However, the underlying mechanism of CTSS upregulation during aging or in abnormal pathological alterations remains unclear. Upregulation of the cysteine protease might serve as scavengers to clear abnormal proteins or peptides (e.g., α-synuclein [50]) that accumulate in the CNS. Nonetheless, dysregulation of homeostasis in CNS microenvironments may further lead to immune and pathological alterations. Although these studies have not directly elucidated the connection between CTSS and cognitive function, the significance of CTSS in cognitive processes cannot be disregarded. In addition, the effects and mechanisms underlying CTSS blockade are well-studied in cancer and inflammation, but the effect of the pharmacological inhibition of CTSS in the brain remains unclear. Here, we determined that CTSS blockade induced hippocampal BDNF mRNA and protein expression and increased synaptic density as well as LTP induction through increasing calcium influx. In the prevailing viewpoints, LTP is attributed to the enhancement of post-synaptic plasticity, promoting branches and reciprocal interactions of neurites [6, 51]. These novel findings indicate that CTSS blockade in the brain induces BDNF expression, promoting neuronal functions. Our data implicated that CTSS might be involved in altering neuronal plasticity. Thus, the suppression of CTSS can improve neuronal functions in older individuals.

Beyond the synaptic function of the neurites, neurogenesis represents another pivotal cascade, involving a series of intricate progressions crucial for strengthening cognitive functions. Neurogenesis is a critical and indispensable process contributes significantly to cognitive functional improvement and memory formation. Interactions between neurons and surrounded microenvironments were integrated during neurogenesis. In this study, we featured the type 2a cells at sub-granular zone (SGZ) of DG, identified through the co-localization of BrdU and sox2 in nucleus, indicating the activation of neural stem cells. Accordingly, BrdU^+^/sox2^+^ cells increased when RJW-58 was presence, suggesting that administration of RJW-58 activating the neurogenesis process. However, since we did not observe the BrdU^+^/NeuN^+^ cells in DG, but the increased expression of DCX^+^ cells in DG supports the postulation of neurogenesis. These results might imply CTSS played a role in maintaining quiescent state of neural stem cells [52]. Intriguingly, despite the enhancement of LTP and BDNF in *Ctss*^*−/−*^ mice, we did not observe an increase in BrdU-incorporated cells, as well as Sox2^+^ and DCX^+^ cells in DG region. Differing from RJW-58 injection mice, the long-term absence of CTSS in *Ctss*^*−/−*^ mice may lead to alterations in the physiological homeostasis in vivo to compensate for its absence. Therefore, progressive changes such as neurogenesis may not be clearly observed in adult unless tissue damage or pathological conditions are induced in *Ctss*^*−/−*^ strain [14, 53]. Interestingly, the amounts of BrdU-incorporated cells in RJW-58 treated mice did not exhibit dose effect as observed in LTP. Neurogenesis provides distinct insights into memory formation by newly born neurons [54–56]. Consequently, the integration of LTP and neurogenesis with reciprocal regulation, might yield synergistic effects within improving cognitive functions. Therefore, we hypothesize that the enhancement of working and spatial memory observed in either RJW-58 injected mice or *Ctss*^*−/−*^ mice was not mediated by neurogenesis, but potentially by LTP and/or BDNF via increasing Ca^2+^ influx. This conclusion is drawn from the consideration that the whole behavior tests conducted did not comprehensively cover the entire neurogenesis period. We postulate that targeting CTSS enhances short-term LTP formation, followed by upregulation of the endogenous BDNF expression. Subsequently, LTP and BDNF served critical roles on promoting neurogenesis.

BDNF is a neurotrophic factor which plays a critical role in CNS. The regulation of BDNF biosynthesis, processing, and release is intricately controlled by rigorous cascades involving both neuronal and non-neuronal lineages [57, 58]. Transcriptional activity of *Bdnf* strongly depended on the Ca^2+^ flow, exhibiting a robust promoting effect on *Bdnf* mRNA for several hours during Ca^2+^ influx [59, 60], indicating the long-lasting features of BDNF signaling triggered by Ca^2+^. In contrast, previous findings have indicated that excessive Ca^2+^ influx in the CNS contributes to the development of neuropsychiatric diseases such as schizophrenia [30, 61, 62]. Dysregulation of BDNF/TrkB signaling might cause the postoperative cognitive dysfunction due to the truncated intracellular domains of the TrkB [63]. Our current work demonstrates that targeting CTSS promotes Ca^2+^ influx and activates BDNF/TrkB signaling through OLF-1, providing a novel connection between CTSS and BDNF/TrkB axis. Also, our finding suggesting that CTSS in CNS functions similarly to calpain, a Ca^2+^ -dependent cysteine protease capable of degrading several substrates including TrkB [64]. Intriguingly, calpain-1 had been reported to exhibit coordinated proteolytic actions with cathepsin B (CTSB) in neurodegenerative disease [65, 66]. The intriguing “calpain-cathepsin” hypothesis proposed calpain-mediated cleavage of carbonylated Hsp70.1 and led to lysosomal permeabilization and/or rupture with the release of cathepsins, and therefore further deteriorated cytoskeleton stabilization of neurons in neurodegenerative diseases [65]. In our study, we demonstrated the efficacy of RJW-58 in promoting BDNF expression, potentially mitigating the dysregulation of calpain-1 mediated TrkB degradation. Therefore, the interaction between calpain and CTSS warrants further investigation.

OLF-1, a dynamic transcriptional activator of the olfactory receptor family in the CNS [67, 68], serves as a regulator of adipogenesis [68] and B-lymphocyte differentiation and is also called “B-cell factor” [69, 70]. OLF-1 participates in epigenetic and transcriptional regulation during B-cell programming [69]. Interactions between OLF-1 and other promoters directly regulate the corresponding gene transcriptional activities [71, 72]. However, OLF-1 cannot bind to DNA and protein simultaneously [68, 73], suggesting that the release of OLF-1 from the protein complex increases its probability to interact with promoters. Our recent study indicated that OLF-1 is involved in IL-10 expression in microglia [18]. In the current study, we observed that OLF-1 bound to the *Bdnf* promoter region once it was released from the OLF-1/CBP/CREB complex. Notably, our findings demonstrate that the augmentation of intranuclear p-CREB was accompanied by an elevation in cytosolic OLF-1 expression, suggesting the increase in free OLF-1 dissociation from CREB in response to CREB phosphorylation. CREB acts as a *Bdnf* transcriptional activator when phosphorylated at Ser133 [30]. Therefore, OLF-1 might serve as another transcription activator of *Bdnf* that co-transcriptionally activates *Bdnf* with CREB*.* Although the biological significance of the extranuclear translocation of OLF-1 remains unclear, studies have indicated that OLF-1 activated survival signaling in GABAergic medium spiny neurons during embryonic neurogenesis [74], suggesting that OLF-1 is required in neuron survival during neuronal differentiation. In addition, the RNA sequencing of *Bdnf* transcripts from HT-22 cells indicates that transcripts were composed of exons I and IX (data not shown) but that the promoter region prediction from BLAST did not match the known promoter regions of exon I, such as USFBE or CRE [75], suggesting that OLF-1 binds to the undetermined promoter region of exon I. Because of the pleiotropic features of OLF-1 in neurons, the intriguing role of this transcription factor in the CNS might be worthy of further investigation.

CTSS is secreted from glial or immune cells and is involved in neuronal activation and synaptic regulation [76]. It exhibits autocatalytic activation at neutral pH, enabling it to be active in extracellular spaces [77]. The role of CTSS in pre- and post-synaptic transmission is intriguing. The current study demonstrates the suppression of CTSS in promoting post-synaptic complexity through increasing calcium influx, shedding light on the possibility of CTSS regulation in pre-synaptic transmission by modulating calcium flux homeostasis in the microenvironment. Furthermore, the release of synaptic vesicles from the presynaptic terminal could be promoted in response to calcium influx through voltage-gated calcium channels [78]. Additionally, CTSS exhibits features of shaping the microenvironment, as evidenced by previous studies suggesting that CTSS digests the peri-synaptic ECM and inhibits dendritic spine density to regulate the circadian rhythm [79]. Therefore, we postulate that CTSS plays an important role in presynaptic neurotransmission and peri-synaptic ECM remodeling. With aging or neurodegenerative diseases, CTSS expression increases in hippocampal astrocytes and microglia [19, 42]. Our results suggest that inhibition of CTSS activity may be beneficial for improving neuronal functions.

## Conclusion

Taken together, the present study suggests that CTSS ablation elevates the intracellular calcium concentration and thereby triggers CaMKII-CREB signaling to enhance BDNF protein expression in hippocampal neurons. Our results also reveal that CTSS ablation significantly increases PSD95 protein levels, dendritic spine density and synaptic plasticity. Our findings contribute to a better understanding of the role of CTSS in the regulation of neuronal function. While it remains to be seen whether these findings have translational validity in humans, our current studies provide new avenue to enhance cognitive function by targeting CTSS.

### Supplementary Information

Supplementary Material 1: Supplementary Figure 1. Targeting CTSS improved learning memory. (A) The diagram illustrates the target hole and nontarget holes in the Barnes maze.  (B) The timeline illustrating RJW-58 administration and the training phase of the Barnes maze for assessing learning memory. (C)(E) Alternations in the mean latency time during the training phase in RJW-58-treated mice (C, *N* = 5 mice / group) and *Ctss*^−/−^ knockout mice (E, *N* = 5 mice/group). (D)(F) Determination of the number of correct visits in RJW-58-treated mice (D, *N* = 5 mice / group) and* Ctss*^−/−^ knockout mice (F, *N* = 5 mice/group) at probe trial-1 and probe trial-2. Bar charts and plots indicate the mean ± SEM. Asterisks indicate significant differences, Sidak’s *post hoc* test in E, Mann-Whitney test in F, *p<0.05,**p<0.01,***p < 0.001 versus the mock group in C and D, versus *Ctss*^+/+^ in E and F. Supplementary Figure 2. Suppressing CTSS delayed spatial memory loss in the Barnes maze. (A)(B) Intragroup analyses of the number of correct visits in RJW-58-treated mice (a, *F*_*mock*_*(3,32)=13.60,p<0.001*; *F*_*2.5mg*_*(3,33) = 1.032,p>0.05; F*_*7.5mg*_*(3,30) = 5.72,p=0.0032*; *F*_*15mg*_*(3,31)= 3.589, p = 0.0246,* *N* = 10 mice/group) and *Ctss*^−/−^ knockout mice (B, *F*_Ctss_^−/−^ (3,12) = 3.738, *p* = 0.0417, *N* = 10 mice) from probe trial-1 to probe trial-4. (C)(D)(E)(F) The distribution of visits of RJW-58-treated mice in the Barnes maze during probe trial-1 and probe trial-2. Bar charts indicate the mean ± SEM.  Asterisks indicate significant differences,Tukey’s *post-hoc* test *p<0.05, **p<0.01, ***p< 0.001 versus probe trial-1 in the corresponding groups in A and B. Supplementary Figure 3. Effects of RJW-58 on intracellular calcium homeostasis. (A) Representative [Ca^2+^]_i_ traces in HT-22 cells in recording buffer containing 1.5 mM Ca^2+^ after RJW-58 induction.  The black arrow indicates DMSO or different doses of RJW-58 added.  (B) Representative [Ca^2+^]_i_ traces in HT-22 cells in Ca^2+^-freerecording buffer after RJW-58 stimulation. The black arrow indicates DMSO or different doses of RJW-58 added. (C) Analyses of dynamic [Ca^2+^]_i_ after RJW-58 stimulation by measuring the AUC.  Quantitative analyses of western blotting for band relative intensity in CaMK IV (D), CaMK IV (E), PSD95 (F, G) from murine hippocampal tissues (*N* = 5 mice/group).  Bar charts in C indicate the mean ± SD, in D, E, F, and G indicate the mean ± SEM.  Asterisks indicate significant differences, Dunn’s multiple comparisons *post-hoc* test in D, E, F, G; *p<0.05, **p<0.01, ***p<0.001 vs the control group in C, vs the Mockgroup in D, E, F, G. Supplementary Figure 4. Supportive data for neurogenesis. (A) The representative images of BrdU-incorporated cells co-express NeuN in the DG of murine hippocampus after 7-day-consecutive RJW-58 injections (BrdU stained green, NeuN stained red). (B)(C) Quantitative analyses of BrdU^+^NeuN^+^ co-expression cells (B), and total NeuN^+^ cells (C) in the DG of different RJW-58 dosing groups (*n* = 6 sections/mouse, *N*= 6 mice/group). (D)(G)(I) The representative images of BrdU^+^Sox2^+^ cells (D), BrdU^+^DCX^+^ cells (G), and BrdU^+^NeuN^+^ cells (I) in the DG of hippocampus of *Ctss*^+/+^ and *Ctss*^−/−^ mice. (E)(F)(H)(J) Quantitative analyses of BrdU^+^ cells (E), BrdU^+^Sox2^+^ cells (F), BrdU^+^DCX^+^ cells (H), and BrdU^+^NeuN^+^ cells (J) in the DG of hippocampus of *Ctss*^+/+^ and *Ctss*^−/−^ mice (*n* = 6 sections/mouse, *N* = 3 mice/strain).  Bar charts indicate the mean ± SEM. NS: non significantly,*p>0.05*, versus mock group in B and C, versus *Ctss*^+/+^ strain in E, F, H, and J. Supplementary Figure 5. Validation of BDNF protein expression level. (A) Quantitative analysis of relative intensity of BDNF in HT-22 cells treated with different doses of RJW-58 ranging from 0 (DMSO) to 5 μM (*N* = 5).  (B) Quantitative analysis of relative intensity of BDNF in murine hippocampal tissues treated with 7-day consecutive RJW-58 injection ranging from 0 (Mock) to 15 mg/kg (*N* = 7 mice/group). (C) Western blot of proBDNF and BDNF proteins in HT-22 cells treated with 2.5 µM RJW-58 for 0 h and 24 h.  (D) Quantitative analysis of relative intensity of BDNF in HT-22 cells treated with 2.5 µM RJW-58 for 0 h and 24 h (*N* = 4).  (E) Characterizations of primary cortical neurons on the tenth day. MAP2 was stained green, and NeuN was stained red.  (F) Western blot indicated that BDNF expression increased in cortex-derived primary cortical neurons from *Ctss*^−/−^ mice than in those derived from *Ctss*^+/+^ mice.  Bar charts indicate the mean ± SEM, Dunnett’s multiple* post hoc* test in A, Dunn’s *post hoc* test in B, unpaired two-tailed*t*-test in d, NS: non significantly, *p<0.05,**p<0.01,***p < *0.001 *vs the control group in C, vs the Mockgroup in D, E, F, G. Supplementary Figure 6. Supportive data of promoter assay and IP experiments. (A) The colored frame highlighted sequences are the predicted OLF-1 binding sites of designated primers in the promoter assay.  (B)(C) Quantitative analyses of relative fold change of OLF-1 (B) and pCREB (C) in the time courses of nuclear/cytosol separation experiments (*N* = 3). (D)(E)(F) Quantitative analyses of relative fold change of CBP (D), CREB (E), and OLF-1 (F) in IP experiments (*N*=3).  Bar charts indicate the mean ± SEM, Dunnett’s *post hoc* test in B and C, unpaired two-tailed *t*-test in D, E, and F, NS: non significantly, *p<0.05, **p<0.01, ***p<0.001 vs the 0 min group in B and C, vs the DMSOgroup in D, E, F. Supplementary Figure 7.  Western blot quantitative data for BDNF/TrkB signaling. Quantitative analyses of pERK (A), and pCREB (B) in cultured HT-22 cells treated with different dosages of RJW-58 ranging from 0 to 5 μM (*N* = 3).  Band intensity analyses of pERK (C) and pCREB (D) along the time axis after RJW-58 treatment (*N* = 3).  Band intensity analyses of BDNF/TrkB signaling including pTrkB (E), pCaMK II (F), pERK (G), and pCREB (H) in cultured primary cortical neurons (*N* = 3). Band intensity analyses of pTrkB (I), pERK (J), and pCREB (K) in single dose of RJW-58 administered murine hippocampus (*N* = 4 / group). Analyses of pTrkB (L) in 7-day consecutive RJW-58 injected hippocampus at the eighth day (*N* = 7 / group), and pERK (M) and pCREB (N) at the seventh week (*N* = 6 / group).  Bar charts indicate the mean ± SEM, Dunnett’s *post hoc* test in A-H, Dunn’s* post hoc* test in I-N, NS: non significantly, *p<0.05, **p<0.01, ***p<0.001 vs the control or DMSO group in A-H, versus the Mockgroup in I-N. Supplementary Figure 8. Supportive data for BDNF/TrkB signaling. (A) Screening of the phosphorylation site of TrkB in response to RJW-58 treatment in the hippocampal tissue.  (B) Western blot analysis of pTrkB and TrkB in murine hippocampal tissues isolated in the seventh week after different dosing treatments of RJW-58.  (C)(D) Western blotting of HT-22 (C) and murine hippocampal tissue (D) for probing pAkt and Akt after designated RJW-58 stimulation for 1 h in HT-22 cells and RJW-58 treatment for 7 consecutive days in murine hippocampal tissues.

Supplementary Material 2: Supplementary Table-1. Antibodies info. Supplementary Table-2. Primer sequences for current study. Supplementary Table-3. Reagents and chemicals.

## Data Availability

All raw data images, and materials that presented in this paper will be made available for reviewers when necessary.

## Abbreviations

CTSS: Cathepsin S
CTSB: Cathepsin B
BDNF: Brain-derived neurotrophic factor
OLF-1: Olfactory receptor transcription factor-1
(EBF-1): (Early B-cell factor-1)
p300: Histone acetyltransferase p300
CBP: CREB binding protein
CREB: CAMP-responsive element binding protein
TrkB: Tropomyosin receptor kinase B
DMSO: Dimethyl sulfoxide
fEPSPs: Field excitatory postsynaptic potentials
LTP: Long-term potentiation
BM: Barnes maze
PTs: Probe trials
BrdU: 5′-Bromo-2′-deoxyuridine
DCX: Doublecortin
Sox2: SRY-box transcription factor 2
CNS: Central nervous system
CaMKII/IV: Ca^2+^/calmodulin kinases II/IV
PSD95: Postsynaptic density protein 95
ERK: Extracellular signal regulated kinase
Tyr: Tyrosine
Ser: Serine
MAP2: Microtubule-associated protein 2
NeuN: Neuronal nuclear antigen (Hexaribonucleotide binding protein-3)
ChIP: Chromatin Immunoprecipitation
IP: Immunoprecipitation
