# Supplementary material for: Targeting cathepsin S promotes activation of OLF1-BDNF/TrkB axis to enhance cognitive function
Source: J Biomed Sci. 2024 May 9;31:46. doi: 10.1186/s12929-024-01037-2 (PMC11084077; doi:10.1186/s12929-024-01037-2)
Supplement: Supplementary file 1 — Supplementary Material 1: Supplementary Figure 1. Targeting CTSS improved learning memory. (A) The diagram illustrates the target hole and nontarget holes in the Barnes maze. (B) The timeline illustrating RJW-58 administration and the training phase of the Barnes maze for assessing learning memory. (C)(E) Alternations in the mean latency time during the training phase in RJW-58-treated mice (C, N = 5 mice / group) and Ctss−/− knockout mice (E, N = 5 mice/group). (D)(F) Determination of the number of correct visits in RJW-58-treated mice (D, N = 5 mice / group) and Ctss−/− knockout mice (F, N = 5 mice/group) at probe trial-1 and probe trial-2. Bar charts and plots indicate the mean ± SEM. Asterisks indicate significant differences, Sidak’s post hoc test in E, Mann-Whitney test in F, *p<0.05,**p<0.01,***p < 0.001 versus the mock group in C and D, versus Ctss+/+ in E and F. Supplementary Figure 2. Suppressing CTSS delayed spatial memory loss in the Barnes maze. (A)(B) Intragroup analyses of the number of correct visits in RJW-58-treated mice (a, Fmock(3,32)=13.60,p<0.001; F2.5mg(3,33) = 1.032,p>0.05; F7.5mg(3,30) = 5.72,p=0.0032; F15mg(3,31)= 3.589, p = 0.0246, N = 10 mice/group) and Ctss−/− knockout mice (B, FCtss−/− (3,12) = 3.738, p = 0.0417, N = 10 mice) from probe trial-1 to probe trial-4. (C)(D)(E)(F) The distribution of visits of RJW-58-treated mice in the Barnes maze during probe trial-1 and probe trial-2. Bar charts indicate the mean ± SEM. Asterisks indicate significant differences,Tukey’s post-hoc test *p<0.05, **p<0.01, ***p< 0.001 versus probe trial-1 in the corresponding groups in A and B. Supplementary Figure 3. Effects of RJW-58 on intracellular calcium homeostasis. (A) Representative [Ca2+]i traces in HT-22 cells in recording buffer containing 1.5 mM Ca2+ after RJW-58 induction. The black arrow indicates DMSO or different doses of RJW-58 added. (B) Representative [Ca2+]i traces in HT-22 cells in Ca2+-freerecording buffer after RJW-58 stimulation. The b [file 12929_2024_1037_MOESM1_ESM.docx]

## **Supplementary Figures**

**
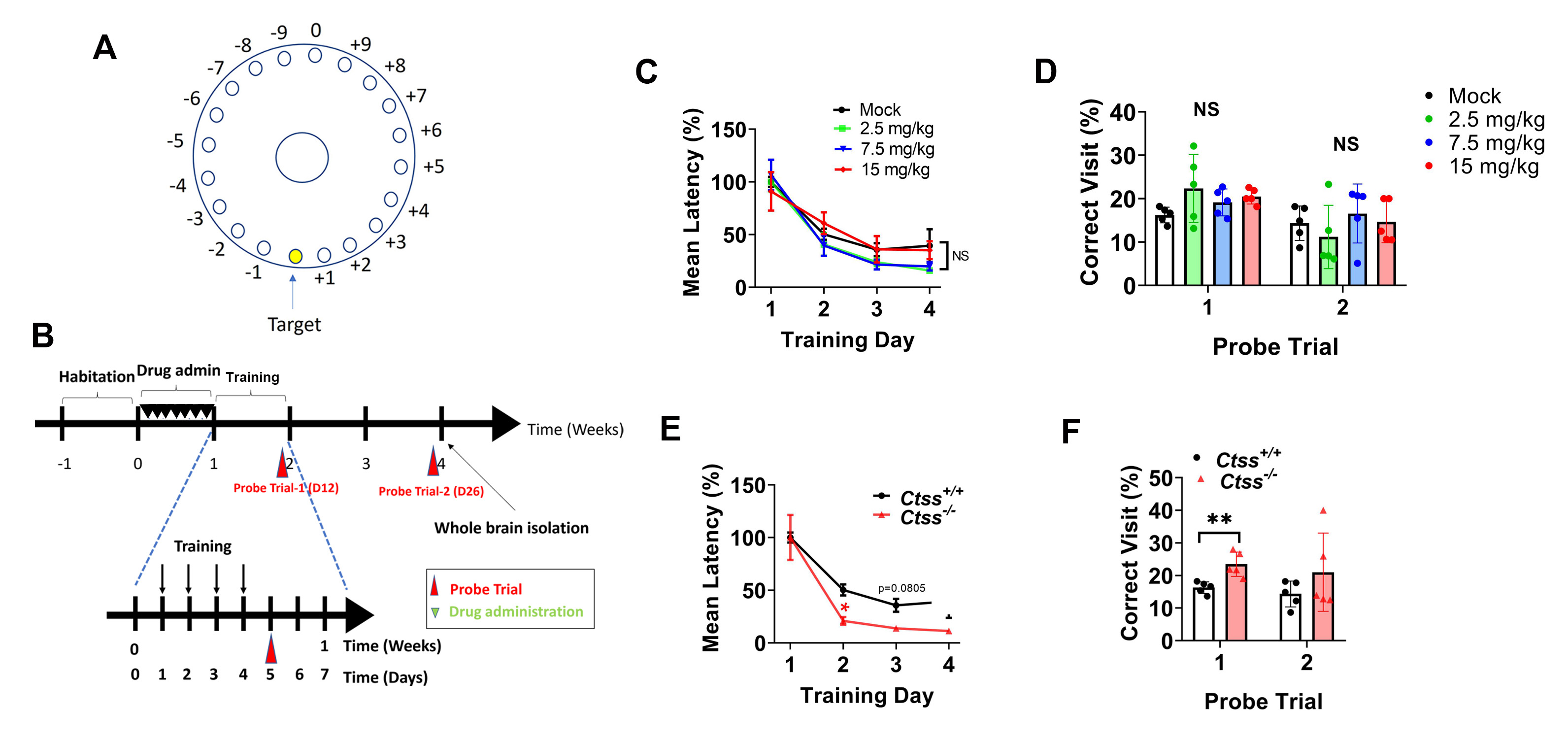
Supplementary Figure-1**

**
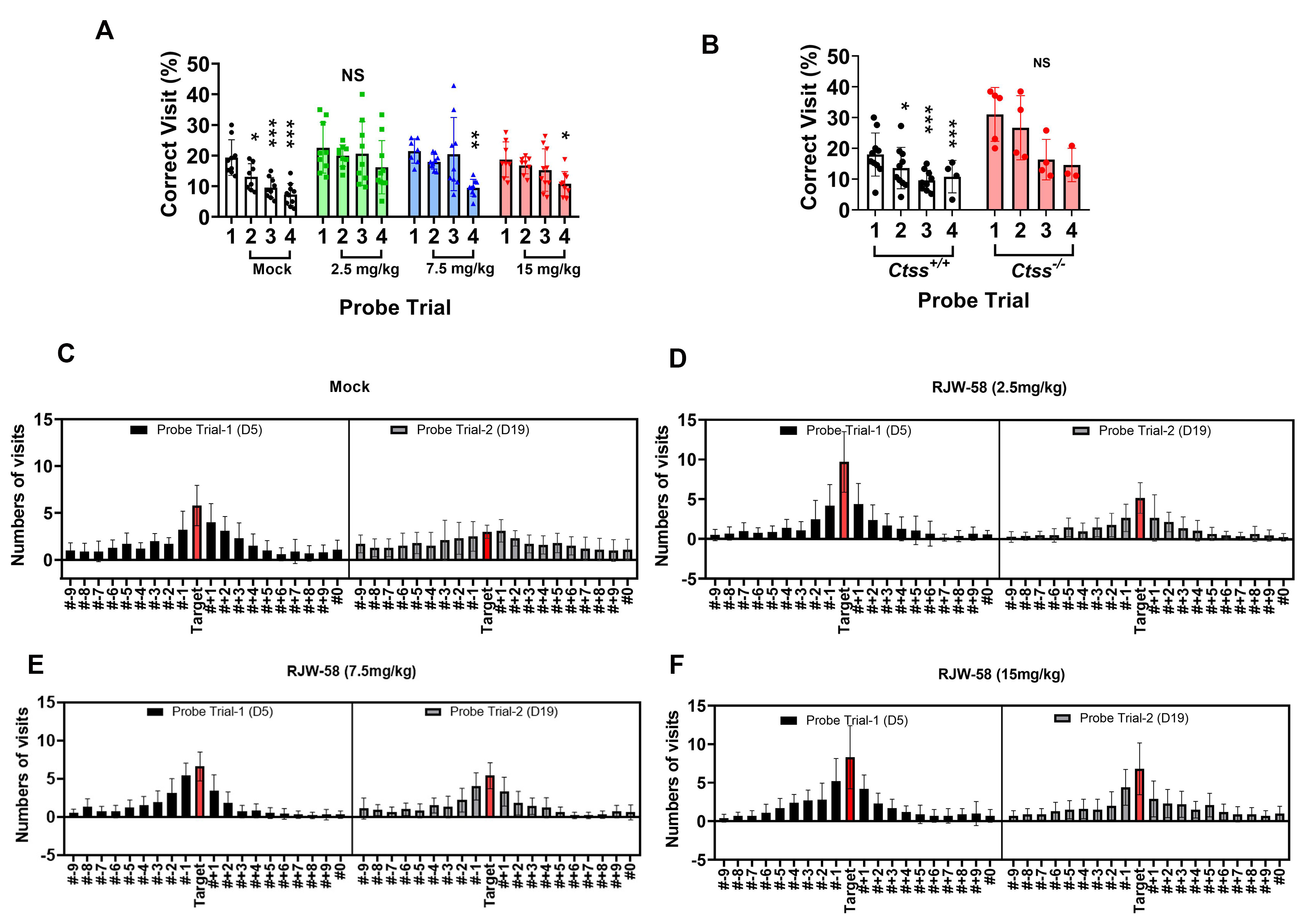
Supplementary Figure-2**

**
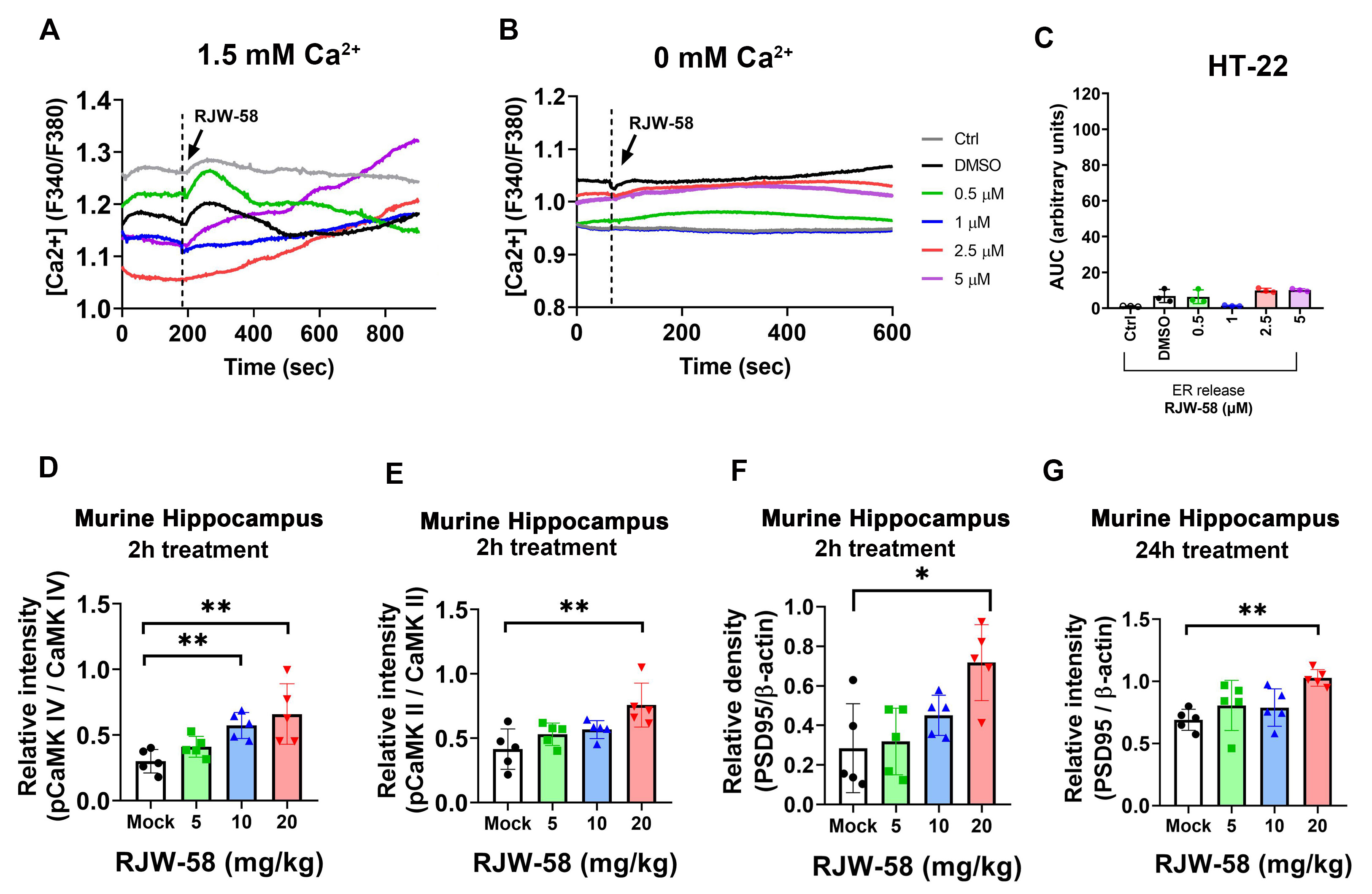
Supplementary Figure-3**

**
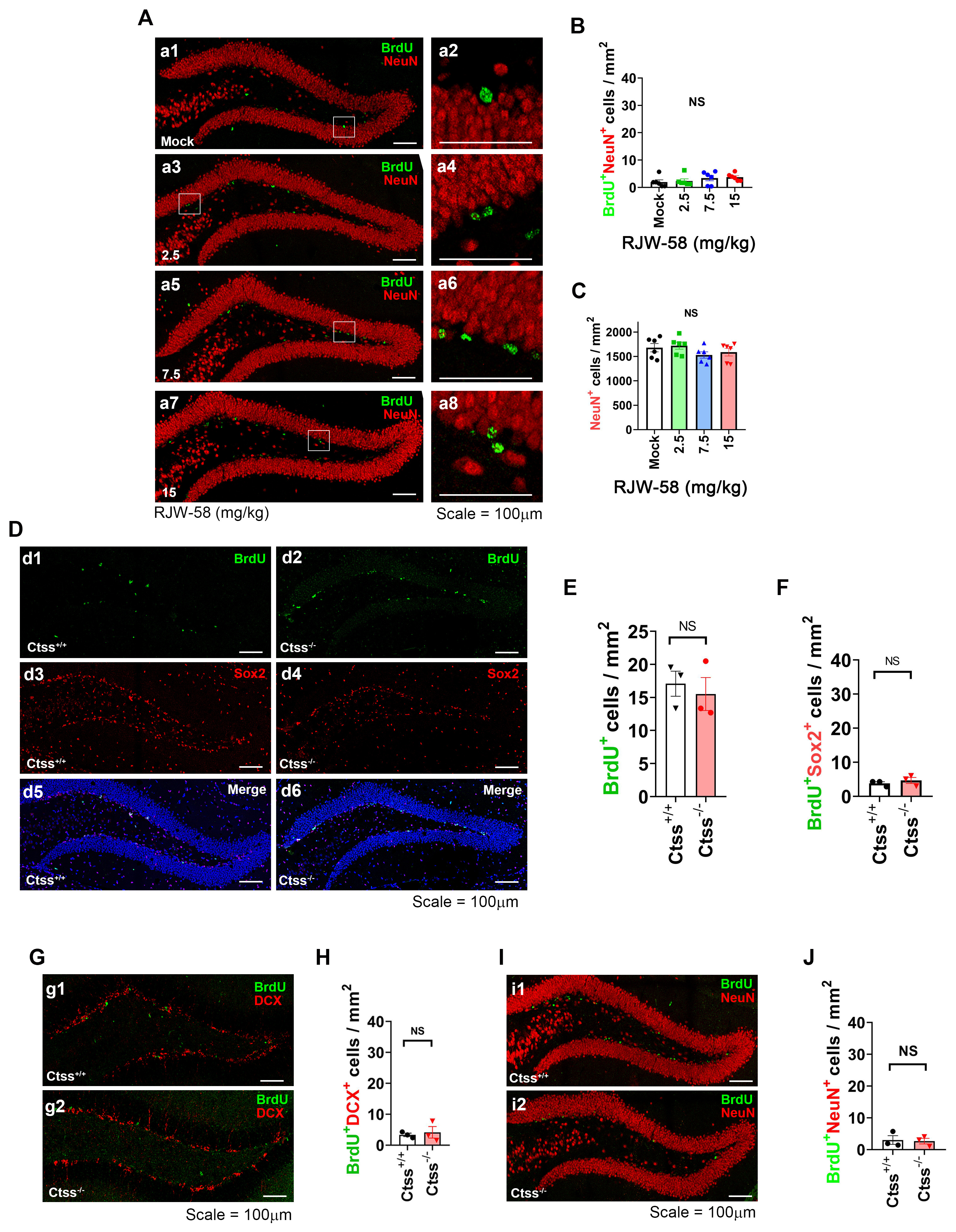
Supplementary Figure-4**

**
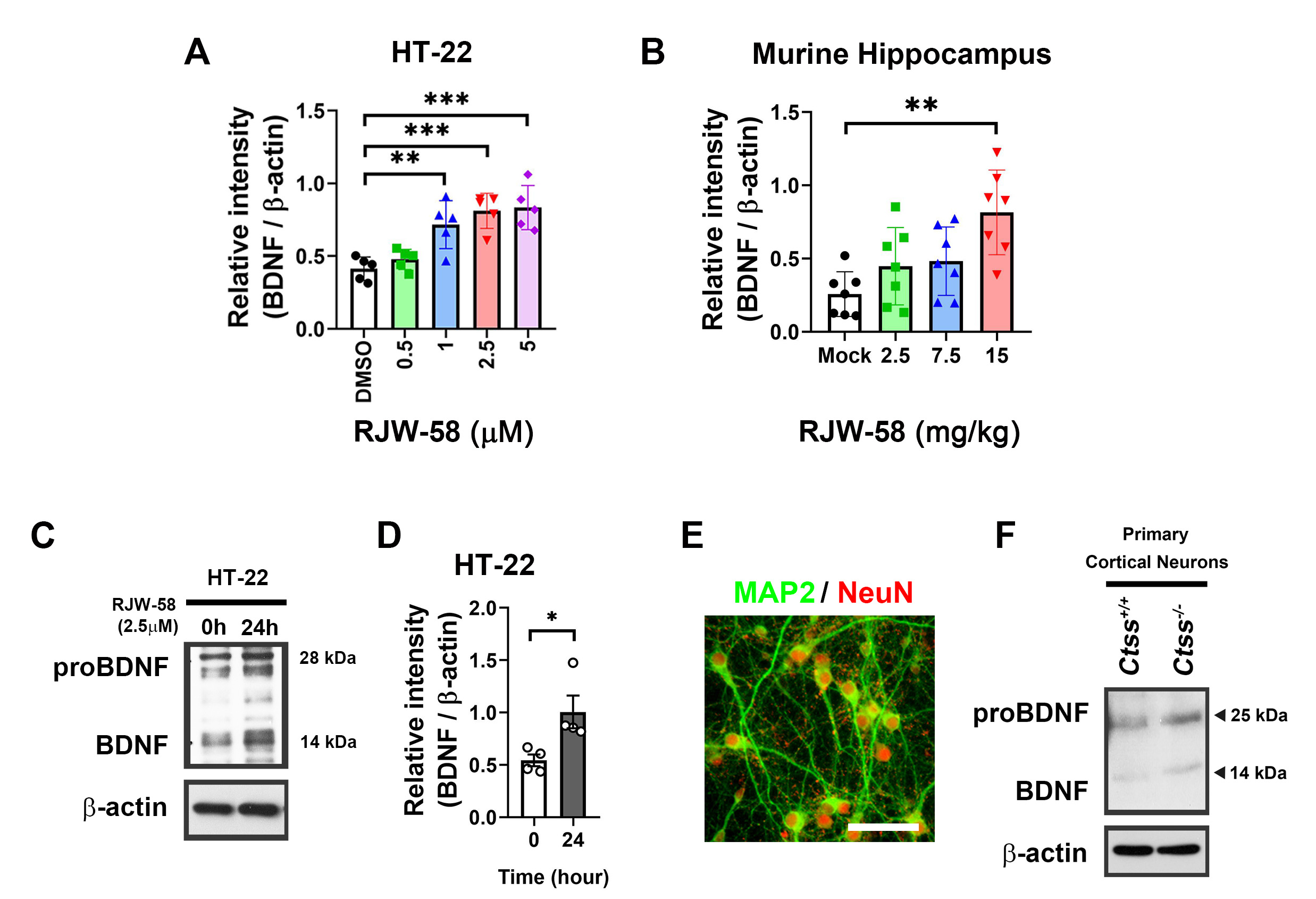
Supplementary Figure-5**

**
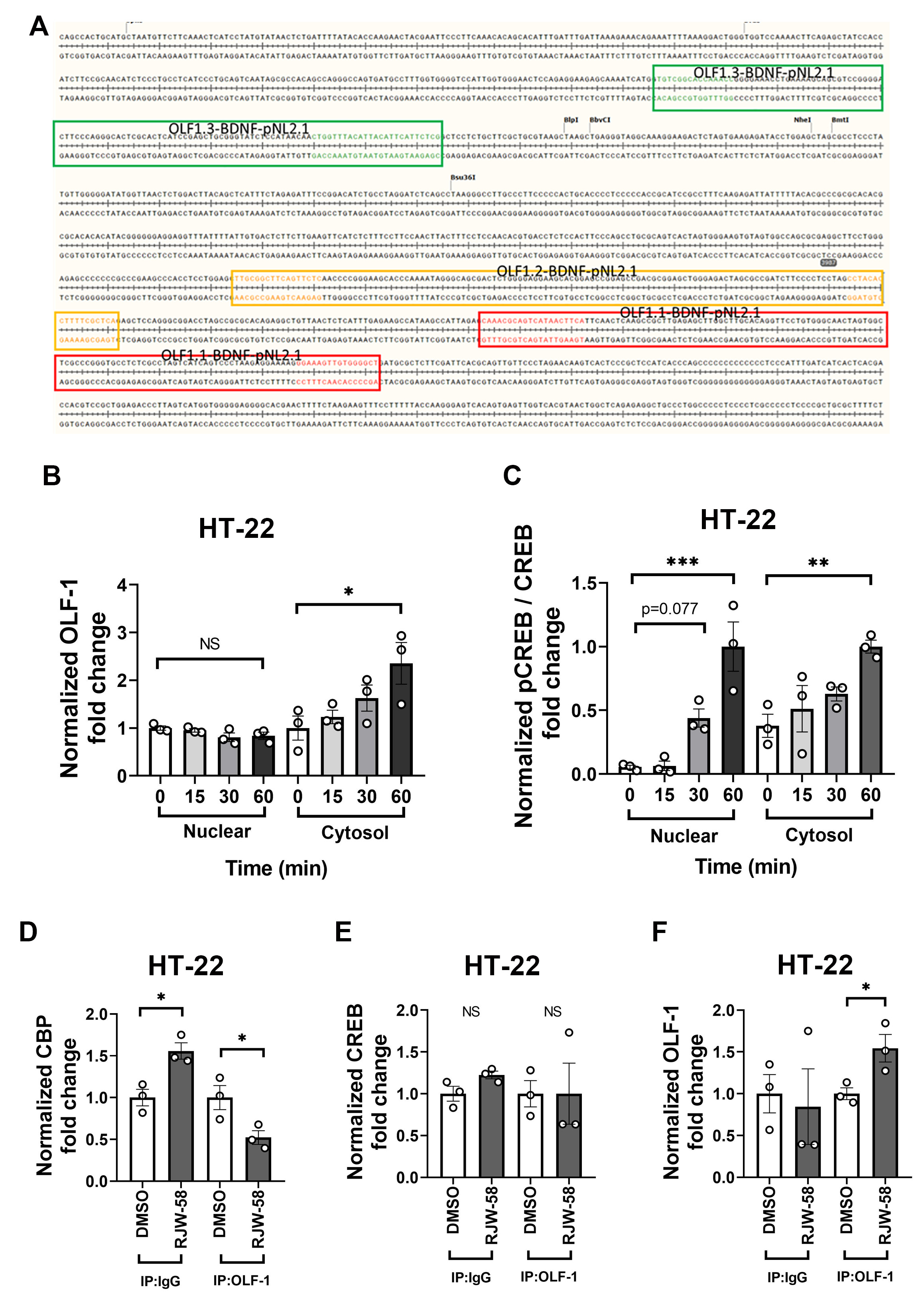
Supplementary Figure-6**

**
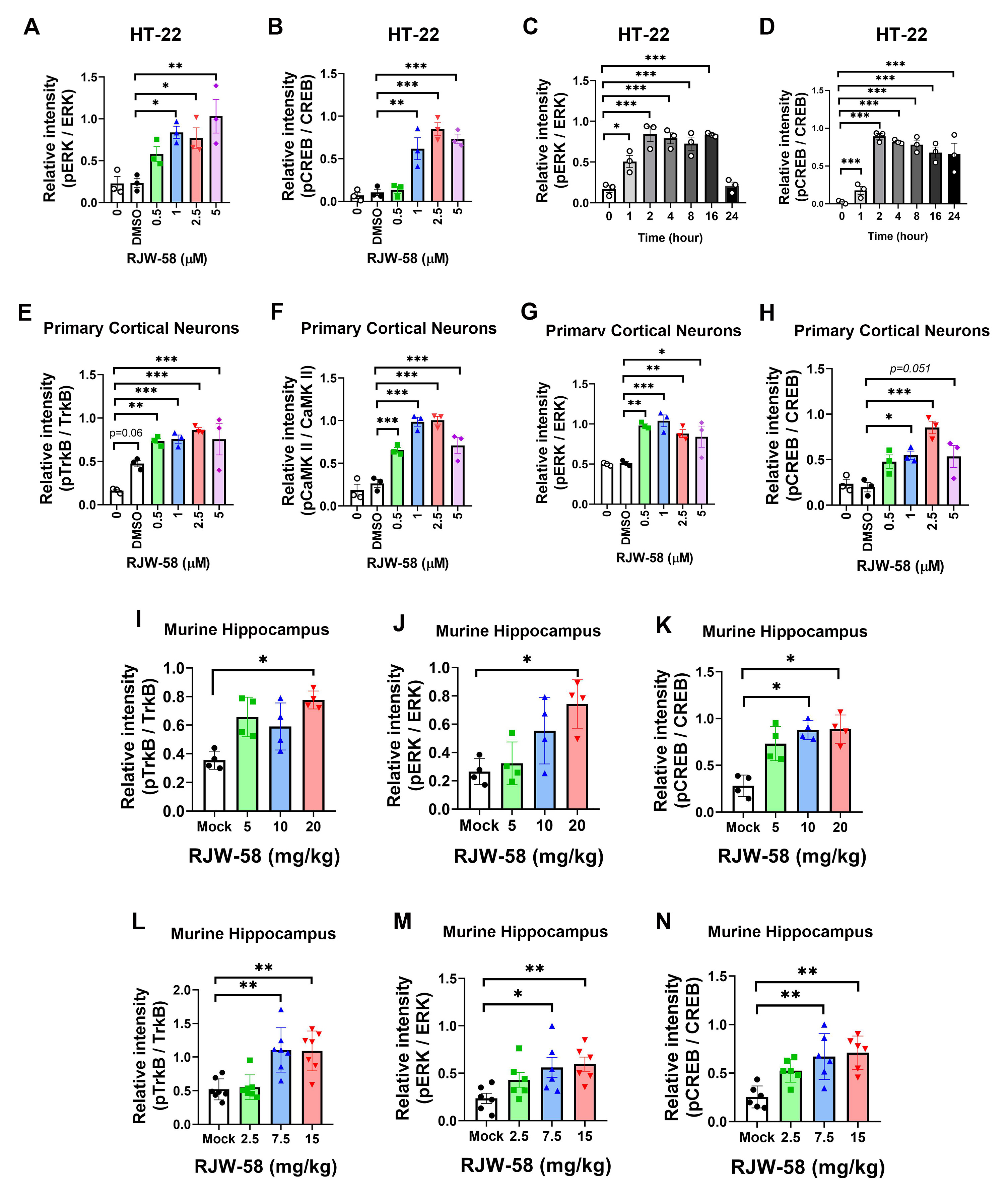
Supplementary Figure-7**

**
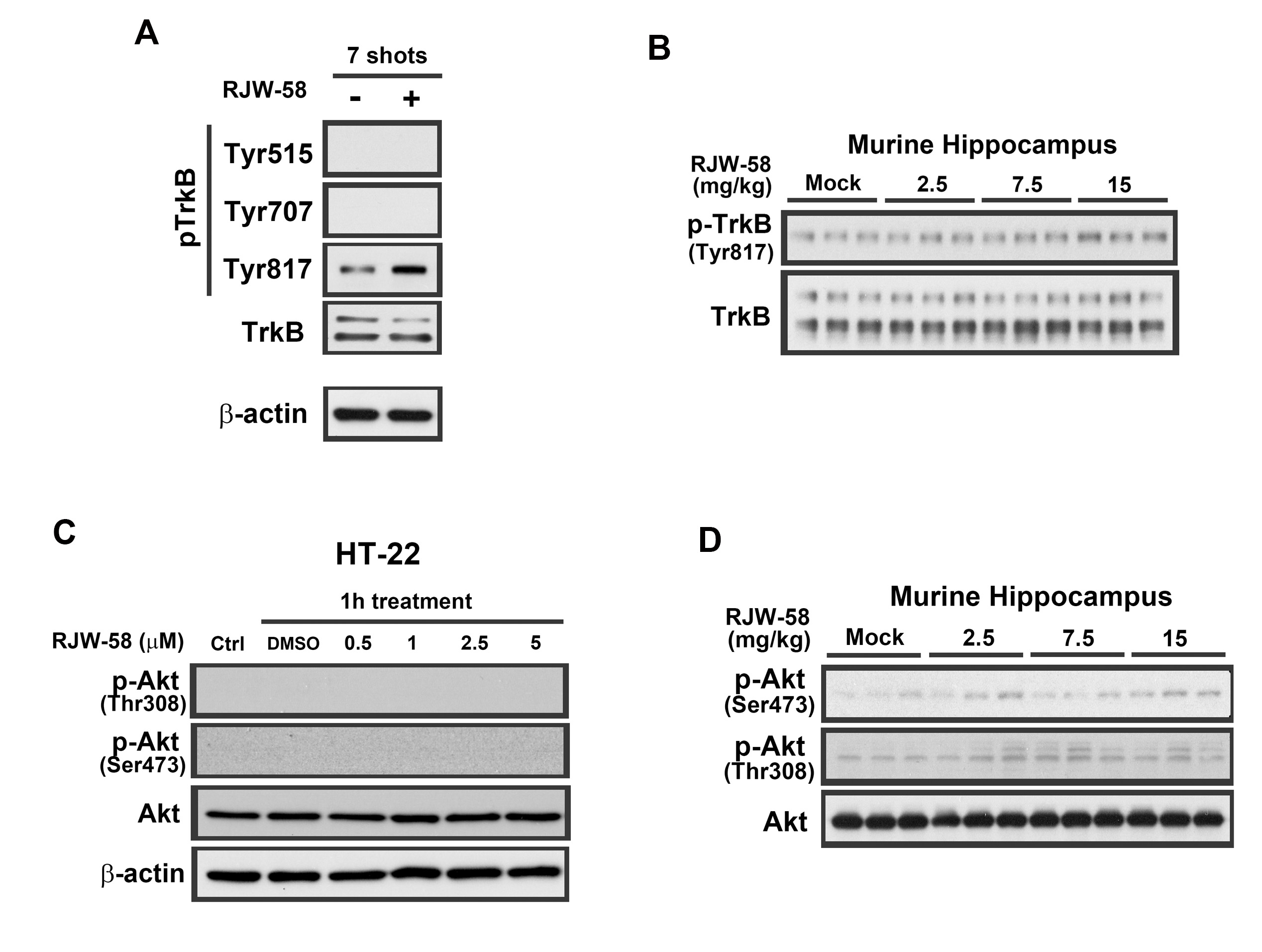
Supplementary Figure-8**

| **Supplementary Table-1. Antibodies info**. | | |  |  |  |
| --- | --- | --- | --- | --- | --- |
| **Primary Antibody** | | **Brands** | **Catalog number** | | **Dilution** |
| p-CaMK II (Thr286) | | Cell signaling | 12716S |  | 1:1000 |
| CaMK IIa | | Cell signaling | 50049S |  | 1:1000 |
| PSD95 |  | Abcam | ab18258 |  | 1:2000 |
| BrdU |  | Novus | NBP2-14890 | | 1:300 |
| Sox2 |  | Abcam | ab79351 |  | 1:200 |
| MAP2 |  | Abcam | ab5392 |  | 1:4000 |
| NeuN |  | Merck | MAB377 |  | 1:1000 |
| p-CREB (Ser133) | | GeneTex | GTX130379 | | 1:1000 |
| CREB |  | Abcam | ab32515 |  | 1:1000 |
| BDNF |  | GeneTex | GTX134514 | | 1:1000 |
| pCaMK IV (Thr196) | | Thermo | PA5-37504 | | 1:1000 |
| CaMK IV |  | Cell Signaling | 4032S |  | 1:1000 |
| OLF-1 |  | SantaCruz | sc-137065 |  | 1:1000 |
| CBP |  | GeneTex | GTX101249 | | 1:1000 |
| p-ERK1/2 |  | Cell signaling | 4370S |  | 1:1000 |
| ERK1/2 |  | Cell signaling | 4695S |  | 1:1000 |
| p-TrkB(Tyr817) | | Thermo | MA5-32207 | | 1:1000 |
| p-TrkB(Tyr707) | | Abcam | ab197072 | | 1:1000 |
| p-TrkB(Tyr515) | | Origene | TA311826 | | 1:1000 |
| TrkB |  | Abcam | ab187041 |  | 1:1000 |
| β-Actin |  | GeneTex | GTX109639 | | 1:5000 |
| Lamin B1 |  | proteintech | 66095 |  | 1:10000 |
| α-tubulin |  | EMD Millipore | 05-829 |  | 1:2000 |
|  |  |  |  |  |  |
| **Secondary Antibody** | | **Brands** | **Catalog number** | | **Dilution** |
| Gt anti-rabbit IgG-488 | | Thermo | A-11008 |  | 1:400 |
| Dk anti-mouse IgG-594 | | Thermo | A-21203 |  | 1:400 |
| Gt anti-chicken IgY-488 | | Thermo | A-11039 |  | 1:400 |
| Mouse IgG-HRP | | GeneTex | GTX213111-01 | | 1:5000 |
| Rabbit IgG-HRP | | GeneTex | GTX213110-01 | | 1:10000 |

**Supplementary Table-2. Primer sequences**

| **Gene symbol** | | **Primer Sequence** |  |  | **Accession number** | |
| --- | --- | --- | --- | --- | --- | --- |
| *RPL13A* |  | F: 5'-CCTGCTGCTCTCAAGGTTGTT-3' | | | NM_009438 | |
|  |  | R: 5'-CGATAGTGCATCTTGGCCTTT-3' | | |  |  |
| *Bdnf* |  | F: 5'-TCATACTTCGGTTGCATGAAG G-3’ | | | LC124913 | |
|  |  | R: 5'-AGACCTCTCGAACCTGCCC-3’ | | |  |  |
| *OLF1.1-pNL2.1* | | F: 5'-CAAACGCAGTCATAACTTCATTC-3' | | | N/A |  |
|  |  | R: 5'-GGAAAGTTGTGGGGCT-3' | | |  |  |
| *OLF1.2-pNL2.1* | | F: 5'-TTGCGGCTTCAGTTCTC-3' | | | N/A |  |
|  |  | R: 5'-CCTACACCTTTTCGCTCA-3' | | |  |  |
| *OLF1.3-pNL2.1* | | F: 5'-TGTCGGCACCAAACC-3' | |  | N/A |  |
|  |  | R: 5'-CTGGTTTACATTACATTCATTCTCG-3' | | |  |  |
| *Ctss^-/-^* genotyping | | F: 5'-GTCAGGCAGATTGCTACAAG-3' | | | N/A |  |
|  |  | R: 5'-ACACTGCTCGGGTGGCAATC-3' | | |  |  |

**Supplementary Table-3. Reagents and chemicals**

| **Item** |  |  | **Brands** |  | **Catalog number** |
| --- | --- | --- | --- | --- | --- |
| DMEM/F12 | |  | Gibco |  | 11320033 |
| Neurobasal-A | |  | Gibco |  | 10888 |
| Fetal Bovine Serum (FBS) | | | Hyclone |  | SH30084.03 |
| L-glutamine | |  | Gibco |  | 25030-149 |
| B-27 supplement | |  | Gibco |  | 17504-044 |
| Penicillin/Streptomycin | |  | Gibco |  | 15140-122 |
| Poly-D-Lysine | |  | Sigma-aldrich | | P6407 |
| Boric acid |  |  | Sigma-aldrich | | 10043-35-3 |
| Borax |  |  | Sigma-aldrich | | 221732 |
| Rapid GolgiStain™ Kit | | | FD NeuroTechnologies Inc. | | PK401 |
| HEPES |  |  | Sigma-aldrich | | H3375 |
| KCl |  |  | Merck |  | 1.04933.0500 |
| Protease inhibitor cocktail | | | Roche |  | 11697498001 |
| RIPA Lysis buffer, 10x | | | Merck |  | 20-188 |
| Bovine Serum Albumin (BSA) | | | Apollo |  | APL-0017 |
| Glycerol |  |  | MDBIO |  | 101-56-81-5 |
| NaCl |  |  | Sigma-aldrich | | 31434 |
| CaCl_2_ |  |  | Merck |  | 1.02382.1000 |
| MgCl_2_ |  |  | J.T.Baker |  | 2444-01 |
| NaHCO_3_ |  |  | Sigma-aldrich | | S5761 |
| NaH_2_PO_4_ |  |  | Merck |  | 1.06346.1000 |
| Glucose |  |  | Sigma-aldrich | | G8270 |
| Kolliphor® EL (Cremophor) | | | Sigma-aldrich | | C5135 |
| TRIzol |  |  | Thermo Fisher | | 15596026 |
| Eukitt mounting medium | | | Fluka Analytical | | 03989 |
| SuperScript III™ kit | |  | Thermo Fisher | | 11736-059 |
| IEM 1754 2HBr | |  | Selleckchem | | S2860 |
| MK-801(+)-Dizocilpine Maleate | |  | Selleckchem | | S2876 |

## **Supplementary Figure Legends**

**Supplementary Figure 1. Targeting CTSS improved learning memory.** (A) The diagram illustrates the target hole and nontarget holes in the Barnes maze. (B) The timeline illustrating RJW-58 administration and the training phase of the Barnes maze for assessing learning memory. (C)(E) Alternations in the mean latency time during the training phase in RJW-58-treated mice (C, N = 5 mice / group) and *Ctss*^−/−^ knockout mice (E, N = 5 mice/group). (D)(F) Determination of the number of correct visits in RJW-58-treated mice (D, N = 5 mice / group) and *Ctss*^−/−^ knockout mice (F, N = 5 mice/group) at probe trial-1 and probe trial-2. Bar charts and plots indicate the mean ± SEM. Asterisks indicate significant differences, Sidak’s *post hoc* test in E, Mann-Whitney test in F, **p* *< 0.05*, ***p < 0.01*, ***p < 0.001 versus the mock group in C and D, versus *Ctss^+/+^* in E and F.

**Supplementary Figure 2. Suppressing CTSS delayed spatial memory loss in the Barnes maze.** (A)(B) Intragroup analyses of the number of correct visits in RJW-58-treated mice (a, *F_mock_ (3,32) = 13.60, p < 0.001; F_2.5mg_(3,33) = 1.032, p > 0.05; F_7.5mg_(3,30) = 5.72, p = 0.0032; F_15mg_(3,31) = 3.589, p = 0.0246,* N = 10 mice/group) and *Ctss*^−/−^ knockout mice (B, *F*_Ctss_^−/−^ (3,12) = 3.738, *p* = 0.0417, N = 10 mice) from probe trial-1 to probe trial-4. (C)(D)(E)(F) The distribution of visits of RJW-58-treated mice in the Barnes maze during probe trial-1 and probe trial-2. Bar charts indicate the mean ± SEM. Asterisks indicate significant differences, Tukey’s *post-hoc* test **p < 0.05*, ***p* *< 0.01*, ****p* *< 0.001* versus probe trial-1 in the corresponding groups in A and B.

**Supplementary Figure 3. Effects of RJW-58 on intracellular calcium homeostasis.** (A) Representative [Ca^2+^]_i_ traces in HT-22 cells in recording buffer containing 1.5 mM Ca^2+^ after RJW-58 induction. The black arrow indicates DMSO or different doses of RJW-58 added. (B) Representative [Ca^2+^]_i_ traces in HT-22 cells in Ca^2+^-free recording buffer after RJW-58 stimulation. The black arrow indicates DMSO or different doses of RJW-58 added. (C) Analyses of dynamic [Ca^2+^]_i_ after RJW-58 stimulation by measuring the AUC. Quantitative analyses of western blotting for band relative intensity in CaMK IV (D), CaMK IV (E), PSD95 (F, G) from murine hippocampal tissues (N = 5 mice/group). Bar charts in C indicate the mean ± SD, in D, E, F, and G indicate the mean ± SEM. Asterisks indicate significant differences, Dunn’s multiple comparisons *post-hoc* test in D, E, F, G; **p < 0.05*, ***p < 0.01*, ****p < 0.001* vs the control group in C, vs the Mock group in D, E, F, G.

**Supplementary Figure 4. Supportive data for neurogenesis.** (A) The representative images of BrdU-incorporated cells co-express NeuN in the DG of murine hippocampus after 7-day-consecutive RJW-58 injections (BrdU stained green, NeuN stained red). (B)(C) Quantitative analyses of BrdU^+^NeuN^+^ co-expression cells (B), and total NeuN^+^ cells (C) in the DG of different RJW-58 dosing groups (n = 6 sections/mouse, N = 6 mice/group). (D)(G)(I) The representative images of BrdU^+^Sox2^+^ cells (D), BrdU^+^DCX^+^ cells (G), and BrdU^+^NeuN^+^ cells (I) in the DG of hippocampus of *Ctss^+/+^* and *Ctss*^−/−^ mice. (E)(F)(H)(J) Quantitative analyses of BrdU^+^ cells (E), BrdU^+^Sox2^+^ cells (F), BrdU^+^DCX^+^ cells (H), and BrdU^+^NeuN^+^ cells (J) in the DG of hippocampus of *Ctss^+/+^* and *Ctss*^−/−^ mice (n = 6 sections/mouse, N = 3 mice/strain). Bar charts indicate the mean ± SEM. NS: non significantly, *p > 0.05*, versus mock group in B and C, versus *Ctss^+/+^* strain in E, F, H, and J.

**Supplementary Figure 5. Validation of BDNF protein expression level.** (A) Quantitative analysis of relative intensity of BDNF in HT-22 cells treated with different doses of RJW-58 ranging from 0 (DMSO) to 5 μM (N = 5). (B) Quantitative analysis of relative intensity of BDNF in murine hippocampal tissues treated with 7-day consecutive RJW-58 injection ranging from 0 (Mock) to 15 mg/kg (N = 7 mice/group). (C) Western blot of proBDNF and BDNF proteins in HT-22 cells treated with 2.5 µM RJW-58 for 0 h and 24 h. (D) Quantitative analysis of relative intensity of BDNF in HT-22 cells treated with 2.5 µM RJW-58 for 0 h and 24 h (N = 4). (E) Characterizations of primary cortical neurons on the tenth day. MAP2 was stained green, and NeuN was stained red. (F) Western blot indicated that BDNF expression increased in cortex-derived primary cortical neurons from *Ctss*^−/−^ mice than in those derived from *Ctss^+/+^* mice. Bar charts indicate the mean ± SEM, Dunnett’s multiple *post hoc* test in A, Dunn’s *post hoc* test in B, unpaired two-tailed *t*-test in d, NS: non significantly, **p < 0.05*, ***p* *< 0.01*, ****p* < *0.001* vs the control group in C, vs the Mock group in D, E, F, G.

**Supplementary Figure 6. Supportive data of promoter assay and IP experiments.** (A) The colored frame highlighted sequences are the predicted OLF-1 binding sites of designated primers in the promoter assay. (B)(C) Quantitative analyses of relative fold change of OLF-1 (B) and pCREB (C) in the time courses of nuclear/cytosol separation experiments (N = 3). (D)(E)(F) Quantitative analyses of relative fold change of CBP (D), CREB (E), and OLF-1 (F) in IP experiments (N=3). Bar charts indicate the mean ± SEM, Dunnett’s *post hoc* test in B and C, unpaired two-tailed *t*-test in D, E, and F, NS: non significantly, **p < 0.05*, ***p* *< 0.01*, ****p* *< 0.001* vs the 0 min group in B and C, vs the DMSO group in D, E, F.

**Supplementary Figure 7. Western blot quantitative data for BDNF/TrkB signaling.** Quantitative analyses of pERK (A), and pCREB (B) in cultured HT-22 cells treated with different dosages of RJW-58 ranging from 0 to 5 μM (N = 3). Band intensity analyses of pERK (C) and pCREB (D) along the time axis after RJW-58 treatment (N = 3). Band intensity analyses of BDNF/TrkB signaling including pTrkB (E), pCaMK II (F), pERK (G), and pCREB (H) in cultured primary cortical neurons (N = 3). Band intensity analyses of pTrkB (I), pERK (J), and pCREB (K) in single dose of RJW-58 administered murine hippocampus (N = 4 / group). Analyses of pTrkB (L) in 7-day consecutive RJW-58 injected hippocampus at the eighth day (N = 7 / group), and pERK (M) and pCREB (N) at the seventh week (N = 6 / group). Bar charts indicate the mean ± SEM, Dunnett’s *post hoc* test in A-H, Dunn’s *post hoc* test in I-N, NS: non significantly, **p < 0.05*, ***p* *< 0.01*, ****p* *< 0.001* vs the control or DMSO group in A-H, versus the Mock group in I-N.

**Supplementary Figure 8. Supportive data for BDNF/TrkB signaling.** (A) Screening of the phosphorylation site of TrkB in response to RJW-58 treatment in the hippocampal tissue. (B) Western blot analysis of pTrkB and TrkB in murine hippocampal tissues isolated in the seventh week after different dosing treatments of RJW-58. (C)(D) Western blotting of HT-22 (C) and murine hippocampal tissue (D) for probing pAkt and Akt after designated RJW-58 stimulation for 1 h in HT-22 cells and RJW-58 treatment for 7 consecutive days in murine hippocampal tissues.
